# Myosin assembly of smooth muscle: from ribbons and side polarity to a row polar helical model

**DOI:** 10.1007/s10974-022-09622-4

**Published:** 2022-07-16

**Authors:** Isabel J. Sobieszek, Apolinary Sobieszek

**Affiliations:** 1 Austrian Academy of Sciences, Dr.-Ignaz-Seipel-Platz 2, 1010 Vienna, Austria; 2 Austrian Academy of Sciences, Vienna, Austria

**Keywords:** Smooth muscle, Myosin filament, Helical model, Building units, Cross-bridge polarity

## Abstract

After decades of debate over the structure of smooth muscle myosin filaments, it is still unclear whether they are helical, as in all other muscle types, or square in shape. In both cases bipolar building units are proposed, but the deduced cross-bridge arrangements are fundamentally different. The opposite polarity of the adjusting longitudinal rows is proposed for the helical structure, while in the case of square filaments, or myosin ribbons, only their two faces are appositively polarized. Analysis of our unpublished archival data on light meromyosin (LMM) paracrystals and myosin rod assemblies as well as the filaments themselves indicated that the rods were assembled with a 6°–7° tilt angle from the rods’ longitudinal axis, in contrast to the lack of tilt in LMM, both exhibiting a 14.3 nm myosin periodicity. Optical diffraction analysis of EM images of the rod assemblies and those of intact myosin confirmed their helical architecture characterized by 28 nm residue translations, 172 nm repeats and 516 nm pitch. A detailed helical model of these filaments was elucidated with bipolar tetramer building units made of two polar trimers. The filaments elongate at their two ends in a head-to-head manner, enabling targeted cross-bridge polarity of the adjacent rows, in the form of a unique Boerdijk–Coxeter type helix, similar to that of collagen or desmin fibers, with the covalent links replaced by a head-to-head clasp.

## Introduction

### Skeletal muscle: stable filaments with ordered structures

The basic unit of the contractile apparatus of skeletal muscle is a sarcomere that is made of two sets of overlapping actins (thin) and myosin (thick) filaments. The thin filaments are composed of actin and associated regulatory proteins and the thick ones consist of myosin. Muscle contraction or tension maintenance results from cyclic interactions between the myosin heads, which project from the backbone surface of the thick filaments, and the thin filaments to convert free energy of MgATP to mechanical work. Evolution has optimized the structure of myosin filaments for most efficient interaction and energy conversion. The thin filaments are mechanically passive, but play an important regulatory role. They are formed from two long chains of globular actin polymers in which the regulatory proteins such as tropomyosin and troponin are incorporated into the double helix of actin filament backbone (Squire 2010).

Under physiological conditions, both types of filaments of the skeletal muscle are stable even though they are composed of non-covalently bound tertiary macromolecular structures; without stable filaments, the crystalline-like architecture of the sarcomeres would not be preserved. Stability of the filaments and their highly-ordered sarcomere assembly made it possible to elucidate the sarcomere and filamentous architectures in the 1960s by the classical electron microscopy (Huxley 1963) and by X-ray diffraction studies (Hanson 1968).

### Smooth muscle: malleable myosin filaments thus the uresolved and controversial fine structures

In contrast to that of striated muscle, the structure of the contractile filaments in smooth muscle remained unclear until the late 1970s, because of the “smoothness” of its intracellular architecture that offers little structural clues (Brading 2006). Perhaps the interest in their structure has been triggered by demonstrations of cross-bridges on isolated smooth muscle myosin filaments (Sobieszek 1972), but certainly, the subsequent seminal discovery of the myosin-linked and Ca/CaM-dependent phosphorylation in the regulation of this muscle by Sobieszek (1977a, b) has boosted the interest. As a result, smooth muscle has become more widely investigated than skeletal muscle, and this has not changed even now.

The difficulties in elucidating the smooth muscle filament structure in general, and that of myosin filaments in particular, have resulted from the high solubility of the myosin, even at physiologically relevant ionic conditions (Hamoir and Laszt 1962). Thus, in early studies the thick, myosin-containing filaments were not observed under relaxed conditions, although the smaller actin filaments were readily observable. As a result, it has been postulated that thick filaments are formed during activation, and before contraction or tension maintenance (Burnstock 1970). This conclusion has been supported by the fact that the 14.3 nm meridional reflections (a characteristic of myosin filaments) were difficult to visualize in earlier X-ray diffraction studies of living smooth muscles and the absence of these filaments in electron microscopy (EM) studies in those years. The 14.3 nm reflection has finally been observed in 1970, under relaxed conditions, and not during contraction or tension maintenance (Lowy and Small 1970). The subsequent demonstration of this reflection under contracting conditions has added confusion about the filaments or their structure, because in contrast to the expectations, intensity of the 14.3 nm reflection was about twofold lower under tension, in comparison to that during relaxation (Vibert et al. 1972; Lowy et al. 1973).

### The ribbons and face polarity hypothesis

The debate over the myosin filament structure became even more complicated and confusing when in an EM study Lowy and Small concluded that myosin elements in smooth muscle possessed the form of ribbons and not the generally accepted round filaments (Lowy and Small 1970). In 1974, the disagreement over the cross-sectional profile of myosin filaments appeared to be resolved in favor of the ribbon shaped filaments (Shoenberg and Haselgrove 1974) but this was not confirmed in a subsequent study (Watanabe et al. 1993). Interestingly, in this study the authors have concluded that during contraction, the number of myosin filaments may increase, but these changes differ in different types of smooth muscle. In the case of rat anococcygeal muscle, the filament number increases with activation, but the number remains the same for guinea pig taenia coli under the same condition.

In a subsequent extensive study of Small and Squire, the ribbon hypothesis was further elaborated by introducing a concept of face polarity, in which the cross-bridges on both ends of the ribbons are polarized in opposite directions (Small and Squire 1972). This has been a logical conclusion for smooth muscles, in which the regular sarcomere-like units are absent, and the myosin ribbons would more efficiently interact with the actin filaments; not only on their two faces but also along their entire length. This apparent advantage has been contested by Sobieszek, one of the authors (from now on referred to as AS), who suggested that round helical filaments could also operate in a manner analogous to that of the ribbons. Instead, he proposed a so called “row polarity” model of cross-bridges within the helical arrangement in which their adjacent rows could be polarized oppositely (Hinssen et al. 1978).

### Square shape filaments of side polarity replaces the ribbons

After successful isolation of longer filaments (up to 8 µm) from isolated smooth muscle cells of taenia coli, the controversy appeared to be resolved in favor of the helical filaments. Specifically, Small has concluded that the ribbons previously observed in EM sections represent an artificial “side by side aggregation” of the round filaments (Small 1977). This conclusion has been further elaborated in a review by these two authors, in which more observations on their mode of assembly and the possible structure are described (Small and Sobieszek 1980).

Nonetheless, in several parallel studies, Craig and his colleagues have incorporated the face polarity feature of the ribbons into their “side polarity” of their square shaped and non-helical filaments. The authors concluded that the cross-sectional profile of smooth muscle myosin filaments is square, and that the cross-bridges at two opposite sides are polarized in opposite directions, the other two sides being free of myosin heads (Craig and Megerman 1977). Although, on one hand this side polarization feature may appear to fit the requirement of the unique structure of the smooth muscle contractile unit, on the other hand it definitely restricts the interaction of myosin heads with surrounding actin filaments. In many smooth muscle types, there can be up to 16 thin filaments surrounding and interacting with a single myosin filament (Sobieszek 1973). In the great variety of different muscle tissues, myosin molecules form the most ordered or even crystalline-like assemblies, the first being demonstrated in the classical EM studies on the insect flight muscles (Reedy 1968). It is therefore not easy to understand why the Craig model of non-helical cross-bridge arrangement appears to be widely accepted in the smooth muscle field, although the round helical filaments appear to be a norm for all other types of muscle systems.

### Invertebrate smooth muscle: a partial breakthrough

Until Lowey and Small’s publication in 1970 (Lowy and Small 1970), it was not possible to see any regular or repeating structures on the filaments isolated from smooth muscles or those formed from purified smooth muscle myosin. Surprisingly, that such an ordered arrangement of myosin heads has been first demonstrated on the filaments formed in crude myosin fraction from vertebrate smooth muscle (Sobieszek 1972) and in thin sections of the molluscan smooth muscle (Sobieszek 1973). After examination of EM images of these self-assembled filaments, the 14.3 nm repeat of myosin has been clearly demonstrated. It results from the regularly spaced crowns of the myosin heads along the entire length of a filament. Thus, there was no cross-bridge free zone in the filament’s center, in contrast to the characteristic feature of skeletal muscle. Instead, there were two asymmetrically located smooth cross-bridge free end-segments for the filaments. These could represent growing or elongating points where polymerization or depolymerization of the filament could occur. From the optical diffraction analysis of the EM images of these filaments it has been concluded that they are assembled into a 6-strand helix of 72 nm repeat with three pairs of myosin heads per crown separated regularly at 14.3 nm (Sobieszek 1972, 1977a). Subsequently, similar filaments from non-muscle myosin have been observed (Hinssen et al. 1978). In more recent reviews, this nanomolar affinity or endogenous association of the smooth muscle regulatory enzymes with this helical filaments have been described (Small and Sobieszek 1980; Sobieszek 1994).

### Breaking free: the proposed helical model

In light of what has been described above, using static light scattering approach, a project has been initiated, aiming to understand the sizes of myosin filaments and their mass equilibria existing during the filament assembly. In particular, it was important to establish what would be a possible building unit of the filament and how this would fit to the previously suggested helical structure. During implementation of this project, it has been confirmed that a myosin trimer is the most stable form of myosin under a variety of ionic conditions (Sobieszek 2016). In a follow up study, AS has concluded that this is also true for an anti-parallel myosin hexamer formed from two trimers (Sobieszek 2022, in press). Thus, a hexamer is the building unit of myosin from which the filaments would assemble.

In the present study, assembly properties of the intact vertebrate smooth muscle myosin and that of its rod coiled-coil proteolytic fragments are reconsidered in relation to the assembly and architecture of the myosin filament. At the same time, the hexamer and trimer building units have been tested for their fit into the original helical filament architecture proposed by AS (Sobieszek 1972). As a result, we propose an improved helical model for these filaments in which the unique alternating polarity of the adjacent rows of the cross-bridges was incorporated. The model not only explains the previously described features such as the asymmetry of the initial (or short) assemblies and their elongation at the two opposite ends, but also incorporates some assembly feature of the Boerdijk–Coxeter type of helices (Sadoc and Rivier 1999; Sadoc 2001). At the same time, the malleability characteristic for smooth muscle is preserved. In essence, the original suggestions made by AS some 40 years ago and elaborated later (Small and Sobieszek 1980) remain valid. This unique helical arrangement is similar to that of collagen and keratin fibers, as well as desmin filaments. The latter has been identified in the smooth muscle (Sobieszek and Bremel 1975) making possible its characterization (Small and Sobieszek 1977). Thus, smooth muscle myosin filaments may assemble in a form of the important α-helix, common for fibrous proteins (Truebestein and Leonard 2016) but with the obvious but important difference necessary for the muscle function; specifically the absence of covalent cross-linking within the filament and its building units.

## Materials and methods

### Preparation of embedded tissue

Ultra-thin sections of freshly dissected taenia coli smooth muscle of guinea pig were fixed for electron microscopy (EM). After dissection from the intestine, a ~ 1.5 cm long muscle strip was placed in oxygenated Ringer solution as described in details by Small and Squire (Small and Squire 1972) and placed in a cold room for temperature equilibration. After replacing the Ringer solution with the same solution containing 0.7% DMSO for the next 30–60 min, the muscle was placed in Ringer containing not only DMSO but also 5% glutaraldehyde. After washing, the muscle was cut into small pieces (about 0.3 mm of size) and post-fixed in osmium/dichromate (1% OsO_4_, 1% K_2_Cr_2_O_7_) for about 1 h. From this point, the protocol used was as the one described in details by AS in 1973, including the EM and image analysis (Sobieszek 1973). Micrographs were taken in a Siemens Elmiskop 101 operating at 80 kV with a 50 µm objective aperture and using primary magnifications between 4000 and 30,000.

### Purification of smooth muscle myosin

Most of the optical diffraction patterns used in the present study were formed in a crude myosin fraction (CMF) preparation, which historically represented a breakthrough in the purification of smooth muscle myosin (Sobieszek and Bremel 1975). In the subsequent years, this protocol has been continuously improved and/or updated in the following publications of AS and his colleagues. Although the latest detailed update for gizzard muscle is given (Sobieszek 1994), this review does not include an important improvement made for the pig stomach myosin. This improvement amounts to a simple addition of a small volume of Pefabloc SC protease inhibitor (0.1 µM final), in addition to the 0.25 mM (final) of the PMSF inhibitor, present in all our buffers used during purification and storage. In order to inhibit the proteolytic activity during the long overnight dialysis of the dissolved crude myosin (a 42.5–55% ammonium sulfate pellet directly dissolved in a low ionic strength buffer; not the CMF), we included Pefabloc SC. This additional inhibitor was added into an open dialysis bag, just before the o/n dialysis in a cold room. In all, this purification protocol ensures that purified myosin is not only homogenous like our gizzard muscle, but also that its regulatory light chains do not become proteolytically modified. It has to be pointed out that this kind of purified myosin contained endogenous CaM/MLCKase complex at levels, which could not be detected by SDS-PAGE, even at high loadings. We termed it as “native myosin” because it could be fully phosphorylated at RT, within 10–20 s by a simple addition of Ca and Mg·ATP (Sobieszek et al. 2005a, b).

### Filament assembly

Filaments were formed using our aliquots of the native unphosphorylated myosin suspension stored at − 70 °C, frozen in liquid nitrogen immediately after completion of their preparation. These were initially dissolved at high ionic strength (AA500 solution) up to the required concentration, and clarified by centrifugation. Short filament formation was carried out by direct 10 to 20-fold dilution of the dissolved myosin, while the long filaments were assembled during a slow overnight dialysis to remove high salt concentrations (Sobieszek 1977a) and for more details see Sobieszek 1994).

### Optical diffraction analysis

Optical diffraction patterns from EM images were obtained from their film positives (masks) using a bench diffractometer or by application of the NIH free software ImageJ. The elaborate description of the software can be found in the corresponding manual online. The diffractometer was of non-commercial type, even though it had been assembled years ago. Since its short description has not been given so far, and because we realized now that it was more reliable and perhaps more convenient than the ImageJ software, we decided to describe it below.

The diffractometer set-up was assembled on a long optical bench on which two 10 cm diameter lenses of 1 m focus length were mounted. The light source was a medium power Neon laser and it was placed precisely at the focus pinpoint of the first lens. The middle distance (between the lenses) was about 1 m and corresponded to the wide beam of the parallel light in which the framed masks were kept in the beam. In the focus point of the second lens, the camera body (without objective) was placed with its film plane at the same distance, making possible to observe an actual diffraction pattern. The frame in which a mask was fixed was equipped with an adjustable rectangular small window or slit; whose length and width limited the examined area. After initial approximate setting of this slit, its position in the X or Y direction was adjustable in order to optimize the pattern by its direct observation in the mirror of the camera body. Often the intensity of the laser red light was high and rather variable; therefore, a short series of shots with different exposure time were taken using a camera body without the lens. This setup was equipped with a red filter to protect eyes from the high intensity focused beam during observations.

## Results

### Electron microscopy of vertebrate and invertebrate smooth muscle

During the early years of 1970, not only the fine structure of mammalian smooth muscle but also that of invertebrates remained controversial and while working in the same field, AS was personally involved in these controversies (Sobieszek and Small 1973; Sobieszek 1972). It is not clear whether this stemmed from the difficulties in the preservation of these fine structures in preparation for electron microscopy (EM) or it reflected the physiological malleability of the contractile apparatuses. The controversies were particularly intense in relation to the myosin filament’s structure. The outstanding resolution in the case of molluscan smooth muscle prompted AS to apply a slightly modified preparative procedure (Sobieszek 1973) previously used for mammalian smooth muscle.

**Fig. 1:**
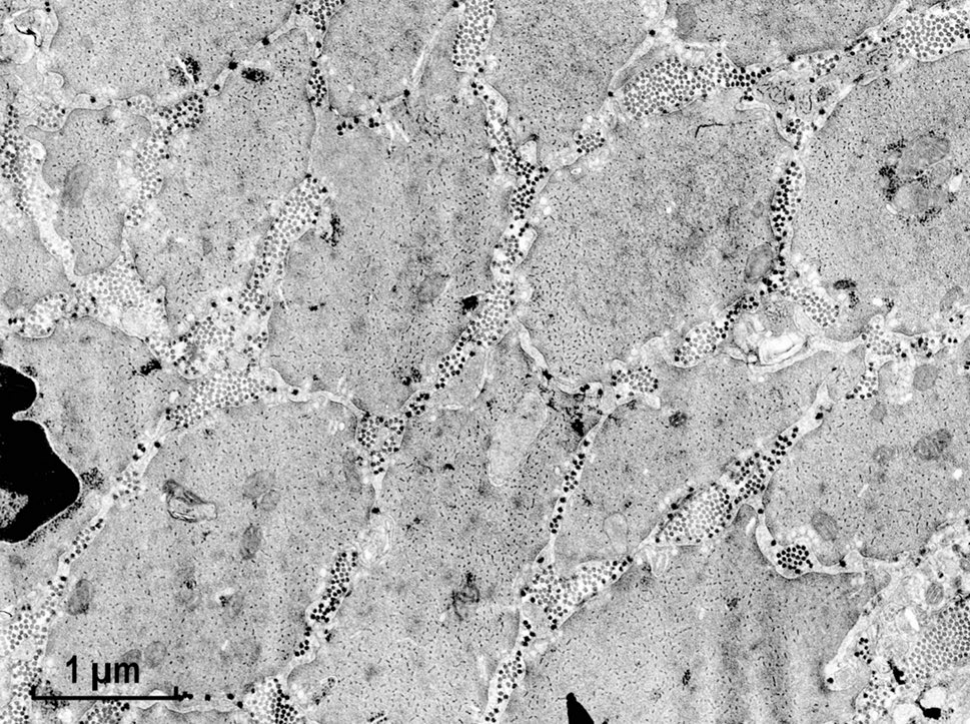
Low power magnification of cross sections of taenia coli muscle. At this magnification, the myosin filaments (some of them exhibiting ribbon-like features) are clearly visible even at 1.5-fold magnification. These shorter ribbons, and not the not recognizable regular arrangements of the actin filaments, later described as actin lattices, are clearly seen in Fig. 3. The applied preparation protocol was as the one used for the molluscan smooth muscle (Fig. 2; see also (Sobieszek 1973)). For more details see “Materials and methods” and “Discussion” sections. Magnification: ×24,000

**Fig. 2:**
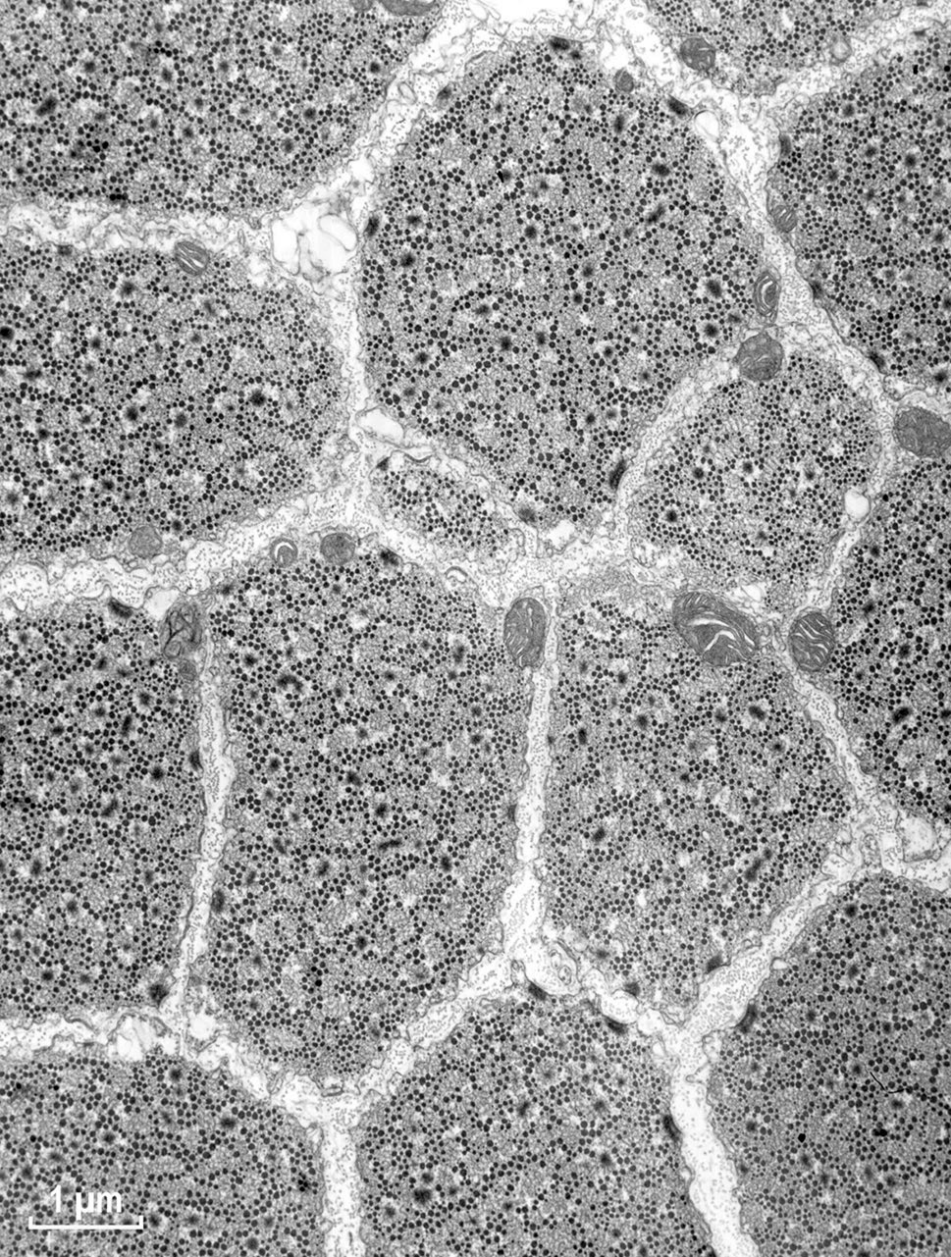
Low power cross section of an EM image of ABRM smooth muscle of Mytilus edulis. The black dots of variable diameter correspond to the myosin filaments, which additionally contain paramyosin and myorod. For more details, see “Discussion” section. Magnification: ×19,500

As shown in Fig. 1, after modification of the protocol (Small and Squire 1972) to the one used by AS (Sobieszek 1973), the preservation obtained for relaxed taenia coli smooth muscle was of good quality and comparable to that obtained for the molluscan ABRM (Anterior Byssus Retractor Muscle) (Fig. 2). Thus, as expected, the structure of the former mammalian smooth muscle appeared uniformly well preserved in the EM cross sections at low magnification (Fig. 1). At this magnification, myosin filaments of the vertebrate muscle are still recognizable, while those of the invertebrates are readily seen because of their huge (up to fourfold) and variable diameters (Fig. 2). This is the characteristic difference between these two types of smooth muscle in their relaxed state.

**Fig. 3:**
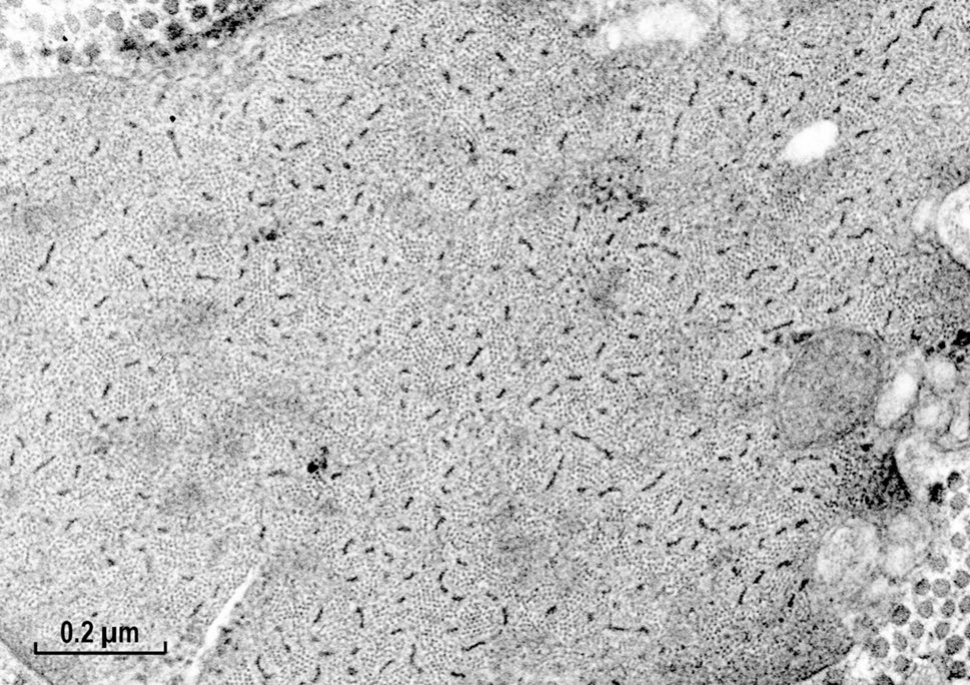
Smooth muscle cell of taenia coli at high magnification. Note, the clear presence of the actin filament lattices. In contrast, most of the myosin filaments are not round but in the form of a ribbon-like shape of variable width. Magnification: ×107,500

**Fig. 4:**
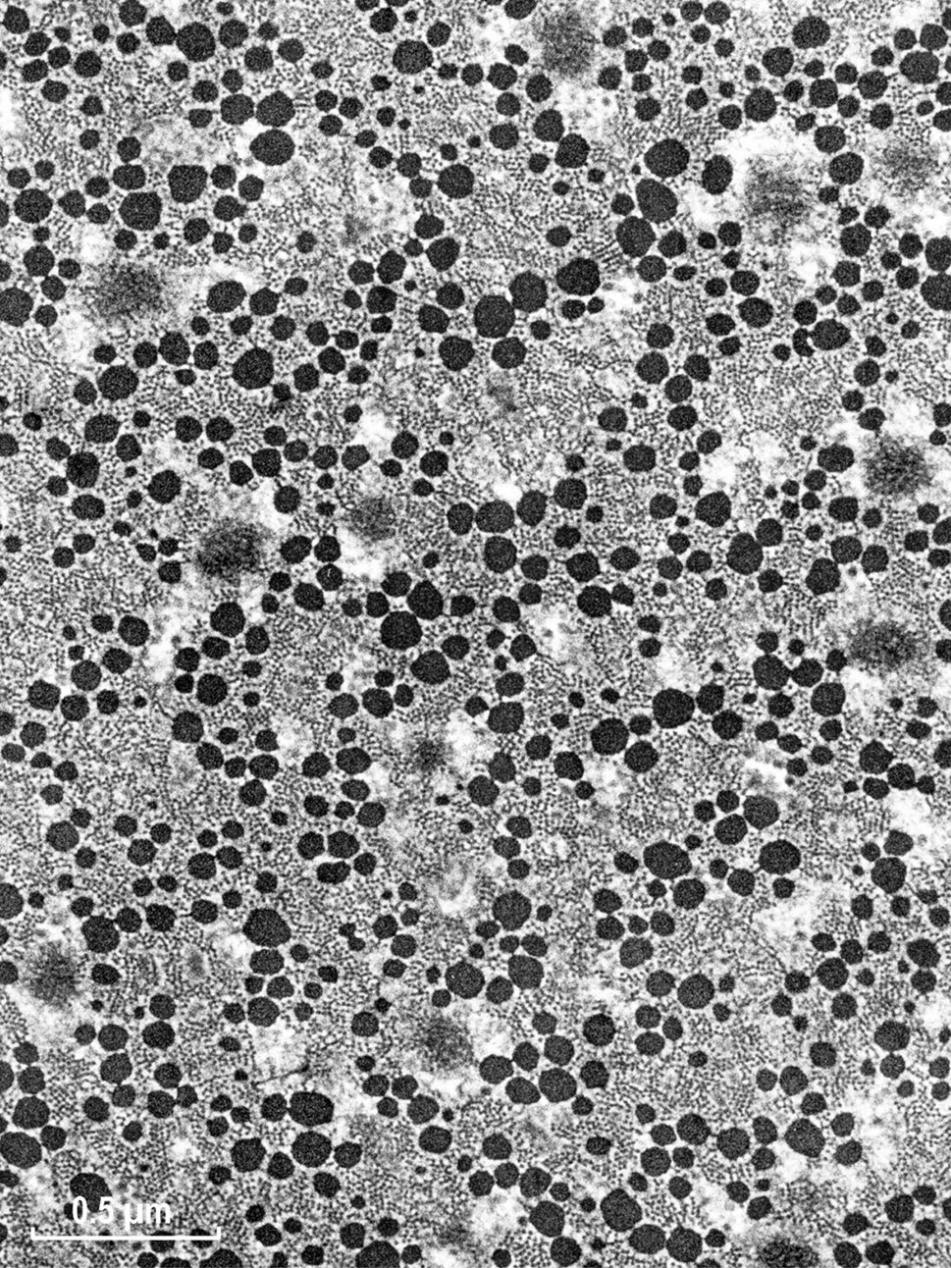
Cross section of molluscan smooth muscle. At this magnification, the myosin cross-bridges are often recognizable as projecting away from the thick filament’ surfaces or connections between the two kinds of actin filaments. The dense areas are structures analogous to the Z-line of skeletal muscle, also present in the vertebrate smooth muscles. Magnification:×63,000

At high magnifications, regular arrays of the thin actin containing filaments were clearly observed within a cell for both muscle types (Figs. 3 and 4). The high degree of order was expected from the presence of the 12 nm X-ray equatorial reflection in the living muscle, which has been preserved until the post-fixation by osmium oxide (see “Materials and methods” section).

Preservation of the myosin filaments in cross sections appeared excellent only for the molluscan muscle (Fig. 4) and it was rather poor for the vertebrate muscle (Fig. 3). Although many well preserved (round) filaments could also be identified, the majority of myosin filaments were of irregular shape, not necessarily being ribbon-like as those observed in the studies by Small and his colleagues (Lowy and Small 1970; Small and Squire 1972). Therefore, the hope for clarifying the “ribbon controversy” was not achieved by this improved preservation and our corresponding EM data were shelved. The controversy remained for years, possibly due to the X-ray diffraction studies (Lowy et al. 1973; Shoenberg and Haselgrove 1974), interpreted as being more consistent with the myosin ribbons, while disregarding well documented conclusions for the filaments in the follow up study of Somlyo group (Ashton et al. 1975).

### Myosin light meromyosin paracrystals

It is the elongated rod or the coiled-coil part of the myosin that appears to be responsible for the assembly of the myosin into filaments. Therefore, before considering the filaments’ architecture, it was necessary to examine the aggregation properties not only of the rod but also of its shorter fragments. A specific proteolytic digestion of purified myosin by trypsin, produces two fragments: heavy meromyosin (HMM) and light meromyosin (LMM). In analogous digestion of myosin by papain, the myosin heads are cut off from its coiled-coil rod, or myosin rod portion. Both sub-fragments are readily soluble at high ionic strength and formed aggregates, which precipitated after dilution with water or dialysis, similarly to the filament assembly of intact myosin. In comparison to intact myosin, their aggregation required a slightly more acidic pH (see “Materials and methods” section).

**Fig. 5:**
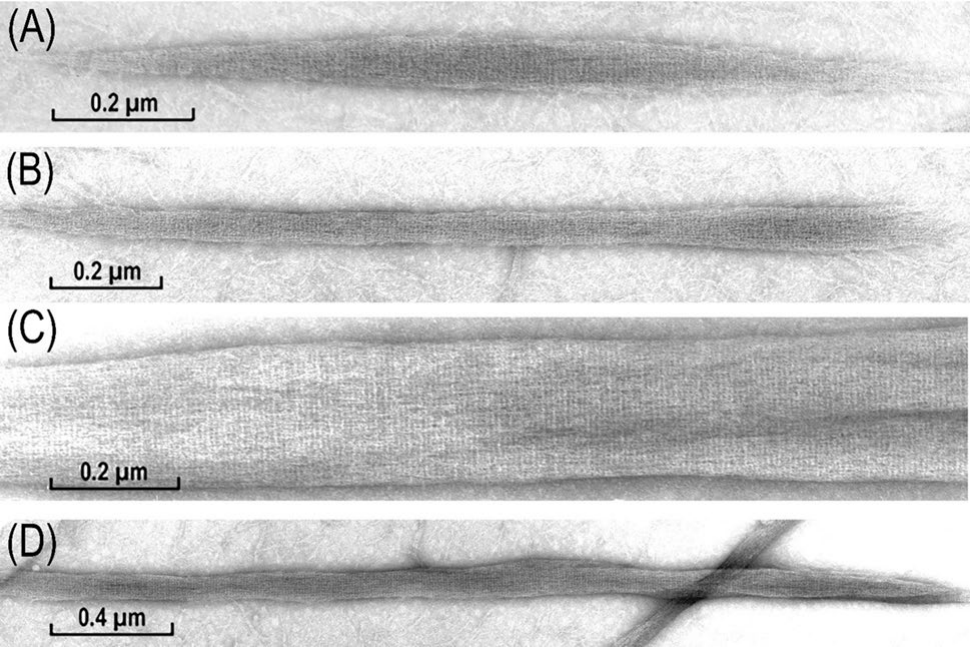
Paracrystals formed from purified light meromyosin fragment (LMM) of chicken gizzard myosin at low acidic conditions. Most commonly they were of the size shown in (**B**), but often they could be relatively broad (**C**) or rather long (**D**). Those in form of a short filament were very rare (**A**). Note the presence of the 7.15 nm striation and the corresponding 14.3 nm so-called leucine repeat resulting from the coiled-coil structure of the LMM and their regular longitudinal assembly. This periodicity was at 90° to the paracrystals axis, which is clearly visible on the large and flat paracrystal shown in (**B**). Magnifications: **A** × 103,000; **B** ×82,200; **C **×94,700; **D **×45,000

The LMM sub-fragment, being approximately 60% shorter than the myosin rod, did not form filaments but large aggregates of variable lengths and thicknesses. They were regularly ordered longitudinally in two and not three dimensions and therefore they are called “paracrystals”. This order or periodicity was clearly seen in the form of striations after EM negative staining (Fig. 5). The observed 7.15 nm periodicity is related to the coiled-coil structure of the myosin rod. Significantly, and as it is clear from the 7.15 nm repeat being at 90° to the longitudinal paracrystals’ axis, the LMM molecules were parallel to this axis, irrespectively of their length (Fig. 5A) or thickness (Fig. 5B) and this has been confirmed by optical diffraction analysis (see below).

### Myosin rod filaments and their thread-like assemblies

**Fig. 6:**
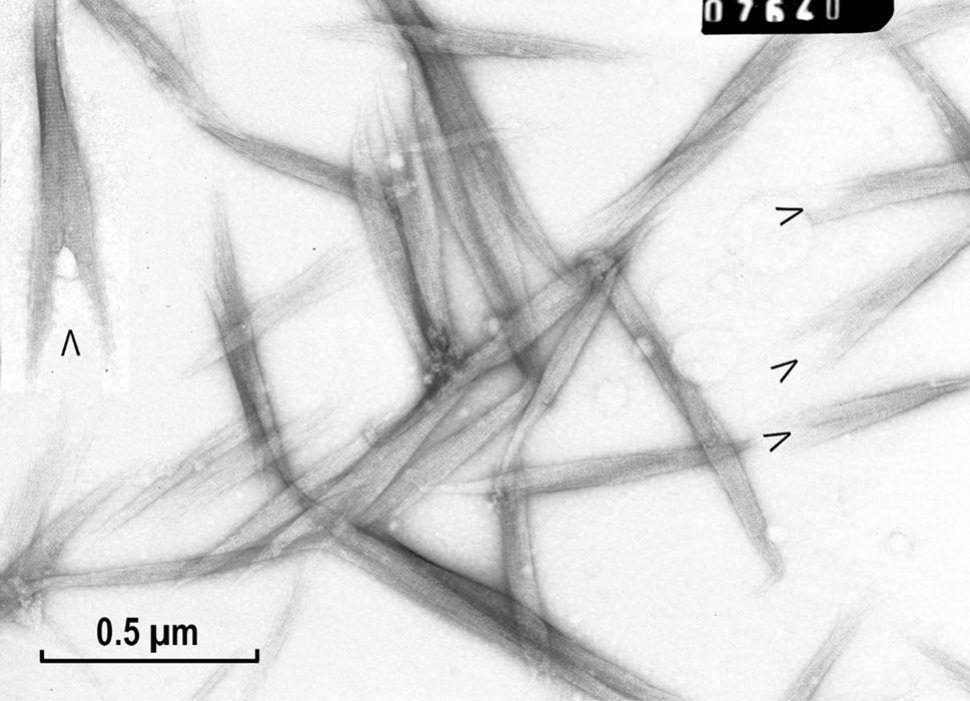
Short filaments or assemblies formed from purified myosin rod fragments at low ionic strength (BW pH 5.6). In these rod filament preparations, there were many assemblies present, in which the filaments were splitting at one end forming arrowhead-like structures (labelled by letter “V”). Note also the presence of the 7.15 nm coiled-coil repeats as observed for the LMM paracrystals. As it is clear from the optical diffraction analysis, this repeat appears to be at about 6°–7° to the longitudinal filament axis. Magnification: ×72,000

In contrast to the LMM fragments, the aggregates of myosin rods resembled these filaments more closely, when they were formed by simple dilution, like those of the intact myosin (Fig. 6). In general, they were shorter than those formed from intact myosin, and did not elongate after slow reduction of ionic strength (see “Materials and methods” section). As shown in the figure, the shorter filaments or aggregates were not uniformly shaped, very often exhibiting an arrowhead like structure (Fig. 6; marked by arrowheads). This interesting shorter form of aggregation must be related to a modification of the assembly (after its initiation), in which these aggregates could not elongate into filaments. Such a splitting was not observed in small-size LMM paracrystals (Fig. 5A), indicating that the packing of the rods was not parallel to the aggregate’s axis. This indicated an involvement of the myosin’s more flexible neck portion (HMM S2 sub-fragment) into the assembly, otherwise appearances shown in Figs. 5A and 6 would be identical.

**Fig. 7:**
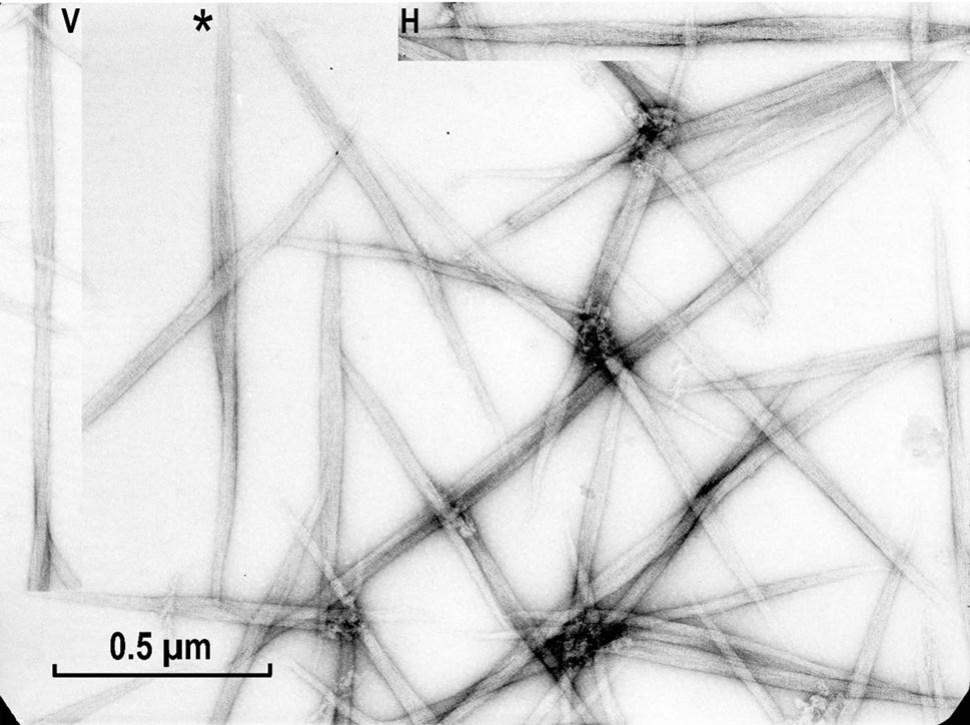
Filaments assembled from purified myosin rod fragment by slow dialysis against low ionic strength buffer at pH 5.6. Under these conditions, javelin-like filaments were formed, exhibiting the same 7.15 nm coiled-coil repeat. Noticeable in their appearance is the somewhat flattened shape, which sometimes resulted in a 90° rotation of the two ends of the javelins in opposite directions. This is clearly seen on the horizontal upper insert (H) and the one labeled with an asterisk. The vertical insert (V) on the left shows an interesting and unique flattened javelin in which three rotated threads are recognizable of the kind shown in Fig. 8. For more details, see “Discussion” section. Magnification: ×72,000

Longer filament-like assemblies of the myosin rod were also formed after very slow dialysis, a method developed by AS (Sobieszek 1977a) for assembly of the long filaments from purified myosin (see “Materials and methods” section). These appeared to have a javelin-like shape (Fig. 7); therefore, they are referred to as rod-javelins or simply javelins. Interestingly, after a closer examination, some of them appeared to be of asymmetrical shape (Fig. 7; marked by asterisk and the insert at the up-left). In this case the non-parallel packing (relative to the javelin’s axis) was more obvious.

**Fig. 8:**
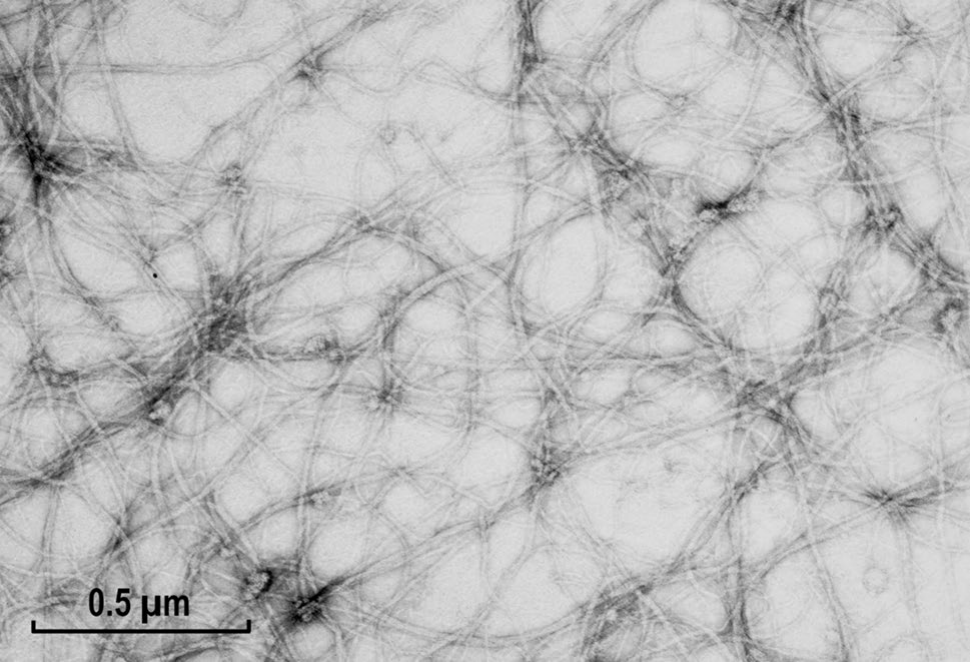
Threads formed from myosin rods after their solubilization at 0.6 M KCl under low acidic conditions (pH about 4.0) with subsequent neutralization (pH 6.8). Note the formation of extremely long thread-like structures, often in form of triple ribbons, which might be related to the coiled-coil structure of the proposed helical thread (see “Discussion” section). Magnification: ×72,000

In contrast to the apparently rigid rod aggregates described above (Fig. 7), under different assembly conditions this myosin fragments also formed flexible thread-like structures. They were formed after their initial solubilization at low pH (about pH 3.5), with a subsequent increase of the pH conditions optimal for the formation of filaments (Fig. 8). Thus, the seeding or initiation of these threads was different from that of the javelins or filaments. These threads were apparently endless because their ends could not be identified, indicating that the flexibility of the rods may be an important factor in the filament assembly. Under these conditions, intact myosin forms short bone-like filaments of constant lengths (see “Discussion” section).

### Self-assembled filaments from purified native-like myosin

**Fig. 9:**
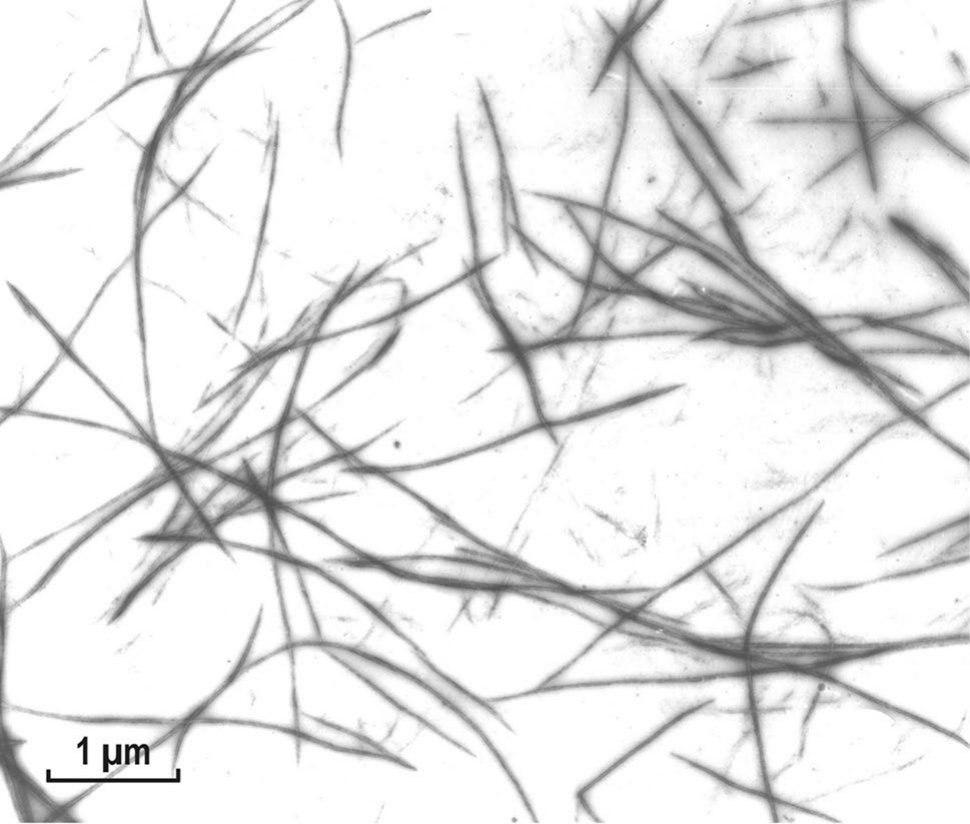
Myosin filaments assembled from purified pig stomach myosin by slow overnight dialysis. These were exceptionally fixed with 1% glutaraldehyde fixation on the EM grid to check whether this would result in their elongation. Apparently, this was not the case because their length was comparable to those of gizzard myosin. Magnification: ×22,000

**Fig. 10:**
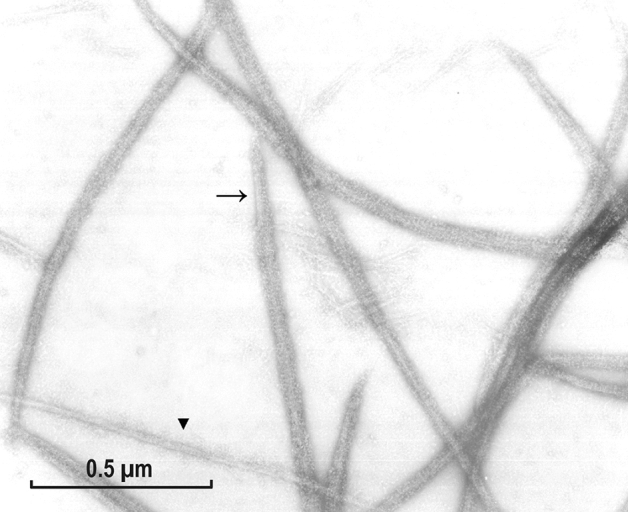
Filaments assembled from pig stomach smooth muscle myosin purified by our improved procedure described in “Materials and methods” section. Note the excellent preservation of the myosin cross-bridges. All the previously described features of such long filaments are clearly seen (see “Introduction” section). This particular EM image was selected because it included an about twofold thinner assembly (at the lower right part of the image) which may correspond to one of the three strands of the supra-coiled-coil assembly proposed (see “Discussion” section). Magnification: ×92,500

After discovering of the smooth muscle myosin phosphorylation (Sobieszek 1977a, b), AS focused all of his work on more important projects related to the Ca/CaM dependent regulation of contraction (see Sobieszek 1991a, b); and on properties of the protein phosphatase involved in their relaxation (see Sobieszek et al. (1997a, b), first identified by Hartshorne group (Shimizu et al. 1994). Nevertheless, he has not given up his interest in the myosin filament structure, and continued recording of EM images and their optical diffraction patterns for future examination. Particularly interesting were the images obtained from his native-like pig stomach myosin. Those were prepared and recorded after successful inhibition of the proteolytic activity during purification of the native-like pig stomach myosin (see “Materials and methods” section). The EM images of such filaments are shown in Figs. 9 and 10 at low and high magnifications, respectively.

As it is shown in Fig. 10, these filaments appeared to be better preserved than those assembled from the gizzard myosin (Sobieszek 1977a, 1972). At high magnification, the regular arrangement of the cross-bridges was clearly visible along the entire filament length except for its smooth edges (Fig. 10, arrow). In comparison with the gizzard myosin, the pig stomach myosin filaments were generally longer, and fewer shorter ones were observed. Significantly, thinner thread-like assemblies could also be detected in these preparations (Fig. 10, triangle). These thread-like structures may be related to their helical structure (see “Discussion” section). We have analyzed scans from these filaments by FFT analysis but the patterns obtained were not as informative than those obtained previously, although the basic 14.3 nm meridional reflection was always present. Perhaps it was due to the higher flexibility of the cross bridges under the conditions used.

### Optical diffraction analysis and deduction of the helical parameters

**Fig. 11:**
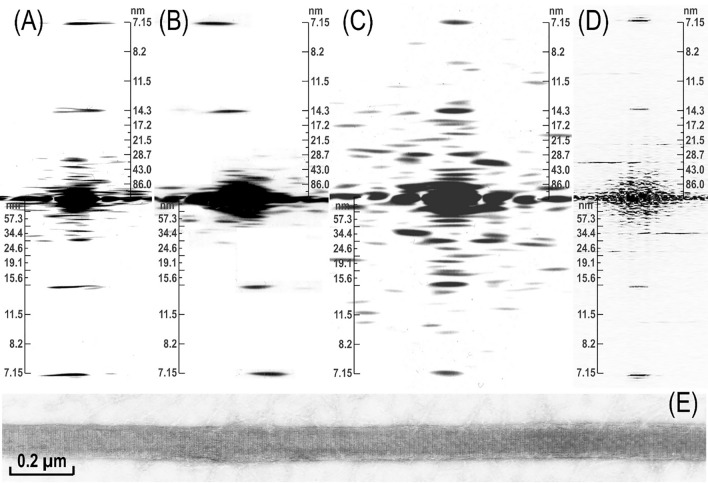
Optical diffraction analysis of LMM paracrystals (**A**), javelin rod filaments (**B**) and myosin filaments isolated from vertebrate (**C**) as well as those of invertebrate smooth muscle (**D**). All were from purified chicken gizzard, except the last one (**D**), which was of Mytilus edulis, and the corresponding EM image used is shown in (**E**). The 7.15 nm meridional reflection is more intense than the 14.3 nm, both characteristic of the coiled-coil structure of the myosin rod. Significantly, in the case of javelins, the 7.15 nm reflection was tilted about 6°–7° relative to the meridian, indicating that the rods were packed at this angle to the javelins’ axis. Note also that in the pattern obtained from intact myosin in **C** there are contributions of the other side of the helix, which results in more symmetrical patterns or individual LLs. The LLs at about 20 and 34 nm are consistent with the 172 nm repeat but not necessarily the 43 nm one. The pattern of (**D**) was an FFT obtained from a scan of the EM image shown below (**E**), using the ImageJ software and not our diffractometer. The FFTs are comparable to those obtained by optical diffraction, except that there were differences due to the large diameter of these molluscan filaments, resulting in a larger number of the cross-bridge rows. Nevertheless, the helical arrangement of the cross bridges is apparent from the presence of the 24 and 36 nm LLs and the 14.3 nm meridional reflection

Analysis of the EM images of LMM paracrystals (Fig. 5) and javelins (Figs. 6 and 7) by optical diffraction showed that they exhibited a strong meridional 7.15 nm reflection, and a weaker one at 14.3 nm, the latter tilted by 6°–7° relative to the javelin’s long axis (Fig. 11B). As indicated above, these two repeats correspond to the coiled-coil structure of myosin, being characteristic of myosin cross-bridge periodicity on the filament’s surface. The tilting indicated that in contrast to the LMM paracrystals, the rods were not parallel to the javelin’s axis. With respect to their structure, a more relevant feature was the presence of a clear layer line (LL) at 72 nm in their optical diffraction patterns (Fig. 11A, B). Weaker LL reflections were also present, among them those at 58 or 43 nm are relevant to the assembly of filaments (see below).

The observation of the 72 nm LL and the 6°–7° tilting indicated that the javelins exhibited not only a helical arrangement but that the actual repeat was even greater than the 72 nm observed before for the myosin filaments (Sobieszek 1977a, 1972). This prompted us to reanalyze all the previously obtained optical diffraction data, together with more recent ones obtained from the pig stomach myosin filaments. Unfortunately, this could only be analyzed from scans of their EM images made just before the retirement of AS. These turned out to be not as informative as those obtained from the film positives prepared for the optical diffractometer (see “Materials and methods” section).

**Fig. 12:**
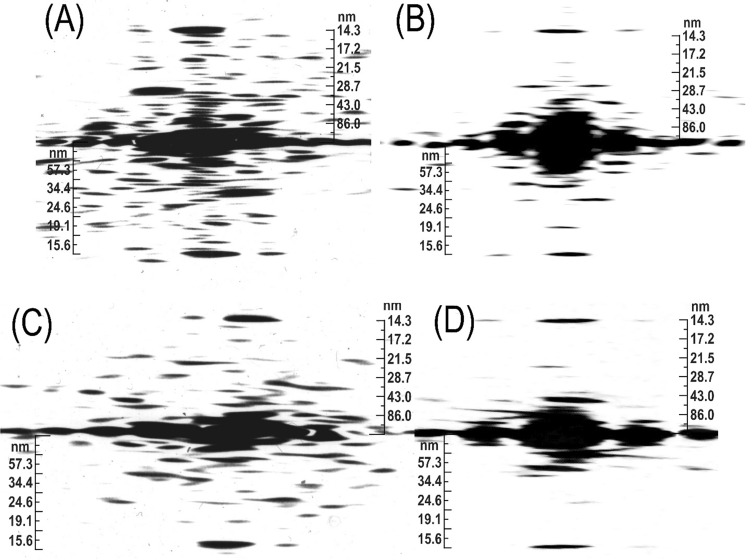
Optical diffraction patterns from myosin filaments (**A**) and (**C**). The patterns (**A**) and (**D**) are perhaps characteristic of the smooth muscle with numerous LLs, which makes their interpretation rather difficult. Nevertheless, the most intense ones are consistent with the proposed repeat. In (**C**) the symmetrical LL spacing was about 72 nm and the spacing of the other one (connected) to a meridional reflection was close to the 43 nm. This pattern was obtained from myosin filament isolated cells of taenia coli. All LLs at about 86 nm can also be identified in this pattern. In the (**C**) pattern, the 34–36 nm LLs and even the nearer 24 nm LLs are also connected in an interesting doublet

In contrast to the diffraction patterns of the javelins, and as expected for the myosin filaments, the 14.3 nm meridional reflection was the major one (Fig. 12), sometimes the related and weaker 7.15 nm was also present (Fig. 11A–C). The 14.3 nm meridional reflections are due to the crowns (made up the cross-bridges or myosin heads), while the latter reflected the packing of the rods (see above). More importantly, there were also “helical” LLs at 86, 58, 43 and 34 nm present, as well as those previously assigned to the 72 nm repeat (i.e., 36 and 24 nm; Figs. 11C and 12A–C) with several weaker ones that could not be easily assigned. Generally, the patterns were complex, indicating contribution not from a single, but perhaps from a couple of helical repeats, as well as the variable contributions from the more distorted microstructure on the other side of the filaments’ helix. A helical structure can be easily recognized in optical diffraction patterns from a symmetrical distribution of the LLs, i.e.; when there are contributions from both sides of a helix.

As indicated above in the huge thick filaments of molluscan smooth muscle, myosin molecules are located on the surface, which is formed from two kinds of proteins (paramyosin and myorod) that are structurally similar to the myosin rod itself. In contrast to vertebrate filaments, these are extraordinary stable structures and with very clear 7.15 or 14.3 nm repeats (Fig. 11E). Some EM images of such filaments were not analyzed by the diffractometer previously, therefore we decided to analyze them using the ImageJ software. Figure 11D shows an example of the FFT (Fast Fourier Transform) obtained. In this pattern, the 7.15 meridional reflection was the one with the highest intensity, and not the expected 14.3 nm one. Most likely it is because all three components of the thick filaments (paramyosin, myorod and myosin rod) exhibit the same leucine zipper coiled-coil structure. Thus, the relative contribution of the myosin heads in this structure was low. Interestingly, in the pattern (Fig. 11D) there is a weak meridional reflection and a couple of stronger off-meridional ones at 36 nm, almost overlapping with the 36 nm LL. Their approximate horizontal separation of about 28 nm might be related to the cross-bridge rows of the same polarity, while the neighboring rows might be oppositely polarized. This would be expected from the much larger surface of the huge molluscan thick filaments. In agreement with the classical study on this muscle (Sobieszek 1973), the myosin cross-bridge repeat is 14.3 nm and they are arranged helically with 72 nm repeats, as confirmed by the presence of the LLs at 24 and 36 nm in the FFT pattern (Fig. 11D). In this pattern the meridional reflection is accompanied by several off-meridional reflections, which were previously used as an indicator of the cross-bridge polarity. No doubt this may be related to the large surface of this molluscan muscle thick filament (for more details see “Discussion” section).

### Helical lattice and the possible filament building units

The structure of the filaments originally proposed by AS in the 70 s is characterized as a three-strand helix with a 14.3 nm residue repeat and a 108 nm pitch that fits best to the 72 nm repeat period (Sobieszek 1977a, 1972). A six-stranded helix with a 28.6 nm residue repeat, and a 3 × 43 nm pitch would also be consistent with the old optical diffraction patterns as shown in Figs. 11A, C and 12A–D, but it would be inconsistent with those obtained from the javelins (Fig. 11B). This indicates that the 72 nm helical lattice did not fit accurately to the observed spacing of these LLs. In contrast, the long-range 58 nm LL and the almost meridional reflection at 43 nm observed for the javelins (Fig. 11B) fitted more precisely to a longer repeat of 172 nm. The patterns obtained from javelins were more precise because they corresponded to the rigid filament backbone, in contrast to the flexible cross-bridges.

On this basis and as it is also apparent from Fig. 12D, we concluded that the actual repeat must be longer, specifically at least 172 nm. The LLs at 28, 34, 43 and 72 nm identified in the diffraction patterns were also consistent with this longer repeat, although these values were not exact. Additionally, the frequently observed LLs at 24 and 36 nm (see Fig. 12C) fitted well the 72 nm repeat. It should be pointed out that actin filaments are also characterized by a 72 nm repeat. Hence, one could expect that some of the free cross-bridges would preferentially adopt a configuration corresponding to their interaction with the actin filaments.

Generally, and as it is obvious from Fig. 11C, even for an almost ideal helical pattern (see above), it was very difficult to make accurate conclusions about the best lattice to use. Therefore, for the rest of the study, the long 172 nm repeat was used as a standard in our numerous fitting attempts. The corresponding helical lattice in its extended form (3 × 172 nm) is shown in the related Figs. 14, 15, 16, 17, and 18. The length of the myosin molecule and its coiled-coil repeat (leucine zipper) agrees approximately with the lattice in its longitudinal dimension, but the diameter was 3 to 5-fold enlarged in these schemes. The extended form of the lattice includes 3 × 172 nm or 36 × 14.3 nm cross-bridge crowns. Importantly, in this lattice the shorter 72 nm repeat is also apparent, perhaps slightly distorted. This not only confirms our previous helical parameters but also contributes to the variability seen in the optical diffraction patterns.

Unexpectedly, at high ionic strength (even higher than that of physiologically relevant) the myosin trimers were the predominant macromolecular form (Sobieszek 2016). Using this approach, the author demonstrated that the largest MW of species eluted from such a column was at least 1500 kDa in size and approximately 45 nm long, which may correspond to an elongated myosin trimer. Thus, it was clear that a trimer or its larger aggregates would be a building unit, which in turn may assemble into a filament. Naturally, the form of the trimer or how the three myosin molecules are arranged within the unit could not be deduced. In the subsequent study using static light scattering approach (Sobieszek 2022, in press), this author characterized fine suspensions of myosin filaments. As expected, and in spite of the high polydispersity of such systems, a frail peak corresponding to the myosin trimer was recognized and this made it possible to assign another peak of slightly higher size to the myosin hexamer. A building unit composed of two hexamers is perhaps too large, and it should be considered as a bipolar “mini filament” (Trybus and Lowey 1987). In turn, three hexamers would form a ring or barrow-like structure and as such could correspond to a seed from which a filament could elongate in two opposite directions (see “Discussion” section).

### A simple transverse and longitudinal packing of the myosin rods

**Fig. 13:**
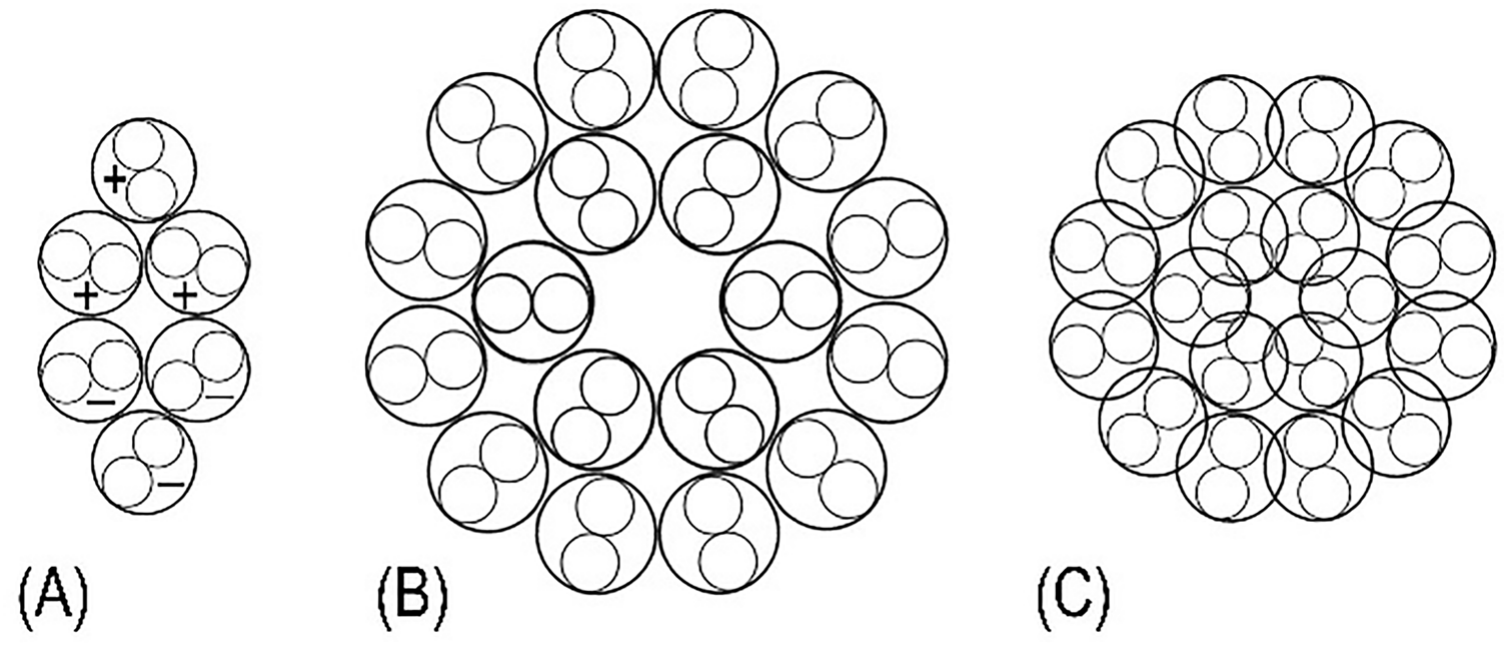
Proposed cross-section of the bipolar hexamer building unit (**A**), the myosin filament (**B**) and as well as its tight form (**C**). Note a high degree of symmetry of the proposed packing of the myosin coiled-coil rods (small inner circles) or their SF-2 sub-fragments. This can be further tightened up to 20% by the proposed incorporation of the SF-2 sub-fragments into the inner ring of the filament’s backbone, possibly resulting in the observed 6°–7.5° tilt. Note in (**A**) that the two trimers are oppositely polarized (marked with + and –), and their basis formed a square in the middle of a hexamer, which was proposed to form the filament building unit and it corresponds to one of the three strands of the proposed helical architecture

We expected that the most probable cross-sectional packing of the coiled-coil rods should be of a high degree of symmetry. A dodecagon composed of myosin trimers in a form of triangles is shown in Fig. 13B. As it is clear from this figure, a tight packing of these rods in the filaments core consists of six trimers assembled into pairs, where each pair corresponds to a hexamer. Thus, the before suggested hexamer building unit fits ideally to this kind of packing of the filament in its cross section. Additionally, the coil-coiled structure of the myosin rod would introduce some flexibility in their packing (Fig. 13C), being consistent with the filament’s dimensions (see “Discussion” section). A building unit or individual coiled-coil helical thread of such arrangement in a cross section is shown in Fig. 13A. There are four possible kinds of helical assembly, which depend on the selected building unit; (i); nine pairs of strands for the bipolar or polar dimers; (ii); six for the trimer; (iii); four for a tetramer, and (iv); three strands for a hexamer (Fig. 13A). After initial unsuccessful attempts to fit a tetramer into the 72 nm helical lattice, we concluded that the third and fourth possibility with the hexamer or trimer are likely the corresponding building units. The question of polarity of the dimeric threads was left open for consideration later on.

**Fig. 14﻿:**
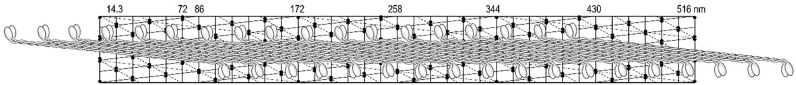
Initial stage of assembly of the bipolar hexamer building units into a helical thread, exhibiting the targeted row variability independent of the overlap length. This kind of asymmetrical myosin assembly has been often observed in the EM images of filaments obtained from crude and purified myosin preparations. The characteristic feature of these shorter asymmetrical filaments is the presence of smooth edges (avoided of cross-bridges) at their opposite ends, resulting from coiled-coil twists of the hexamer building units within a seeding fragment of a helical thread (see Fig. 13A). The relative length of the observed smooth edges is about 1/3 shorter as the twist was not incorporated in this 2D model and its width is even smaller in case of the tight configuration (Fig. 13C). For more details about this kind of assembly and a discussion on how they are formed, see review by Small and Sobieszek in 1980 (Small and Sobieszek 1980)

Independently of the length or the type of the hexamer unit, the assembling process is most easily understood as it is illustrated in Fig. 14. Although in this figure, the overlapping regions of two trimers within a hexamer cannot be identified, the polarity of the cross bridges is easily recognizable. The longitudinal polarity of the cross-bridges within a single helical strand is also recognizable. This kind of asymmetrical filaments has been observed in numerous previous studies but have been differently interpreted (see “Discussion” section).

### Form of the hexamer unit and myosin assembly

**Fig. 15:**
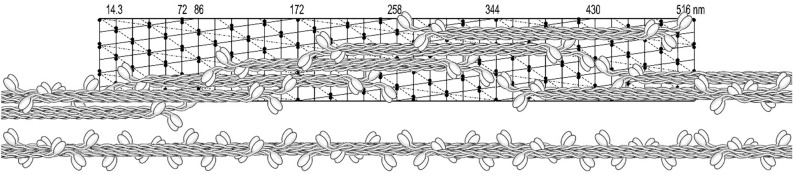
Polarity of the cross-bridges of a helical thread assembled from a simple bipolar hexamer made up of two polar trimers. Note that in this specific arrangement, the groups of 3 adjacent cross-bridges are polarized (or directed) on opposite directions and that the overlap within the hexamer is maximal. Note also that the last 3 or 4 hexamers were not extended upwards but placed below still on the lattice. The assembly of a thread (shown below the lattice) is analogous to that of collagen, where trimeric proto-collagen forms a three-strand coiled-coil collagen fiber (see “Discussion” section). It is important for this figure as well as all figures below, that the angle of a single head pair represents only the direction in which these heads would be pulling the thin actin filaments, but not necessarily the myosin filaments’ polarity

With the known length of the rod (150 nm; see Elliott et al. 1976) and the 14.3 nm repeat between two crowns of the myosin heads, the rod covered 10.5 of the repeats. As it is shown in Fig. 14, the shortest form of a bipolar hexamer unit was 258 nm long and exhibited the maximal 230 nm overlap with the 14.3 nm stagger within its two trimers. Such a unit fitted well to the 172 nm helical repeat and it was convenient in our 2D helical modelling of the supra-coiled-coil filaments’ assembly. In analogy to desmin or collagen fiber formation, these units could assemble and elongate by attaching the units at their two ends while forming long supra-coiled-coils threads. During this elongation, myosin heads at one end at one hexamer unit, have to interlock with the opposite end of the attaching hexamer unit (Fig. 15). Although the coiled-coils threads were readily formed from these units, the polarity of the heads was not exactly of the type which we were aiming for; i.e.: the one originally proposed by AS in 1972 (Sobieszek 1972). Closer examination of the polarity shows that there are sets of three cross-bridges of the same polarity, and then the polarity of a set changes to the opposite, and so on.

**Fig. 16:**
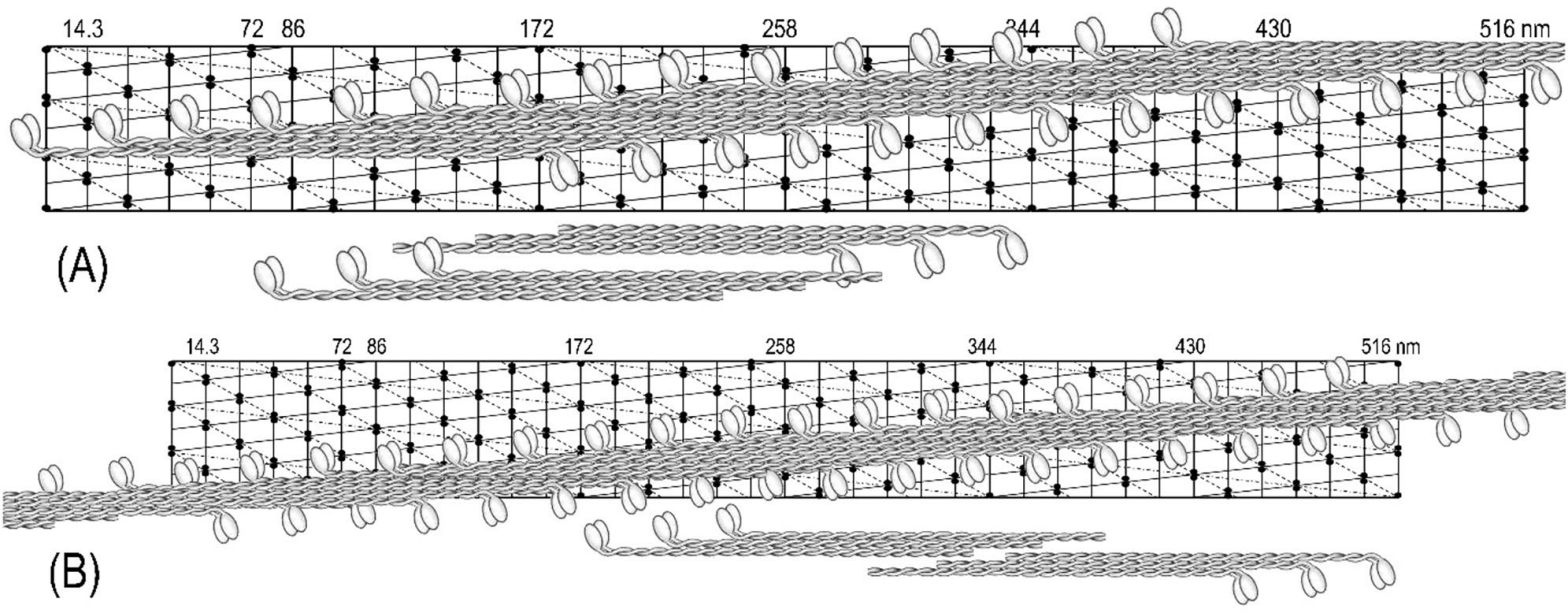
Initial assembly of the hexamer units of shorter and longer overlap into a continuous helical strand. The corresponding units are shown below the lattices in form of separate trimers of 2 × 14.3 nm stagger making possible estimation of their overlap’s regions. A hexamer unit is formed from two anti-parallel trimers shown below and it extends over two helical repeats (2 × 172 nm). Note that the polarity of the cross-bridges continuously changes from right to left, because of the attachment of the two opposite ends of the two hexamers within a single combining period (6 cross-bridge crowns). For more details, see text

There are two hexamer assembly units which can be considered in the 2D assembly on the concluded helical lattice. In contrast to the one used in Fig. 15, the stagger of the two possible trimers (within the hexamer) has to be longer; i.e.: 2 × 14.3 nm and exhibit the same polarity. In addition, and as shown in Fig. 16, the hexamers differ in their lengths; the first (of long overlap; A) is shorter (i.e.: 258 nm) and the other one (of short overlap; B) is 344 nm long. Polarity of the cross-bridges, within a filament assembled from these three building units (including Fig. 16A), would be of the row type as originally suggested by AS (Sobieszek 1972). Specifically, the adjacent longitudinal rows of the cross-bridges would be oppositely polarized in order to pull two singly polarized actin filaments in the opposite direction. Therefore, we propose to term such an arrangement as the targeted row variability of the cross-bridges, and we will use this term from now on.

**Fig. 17:**
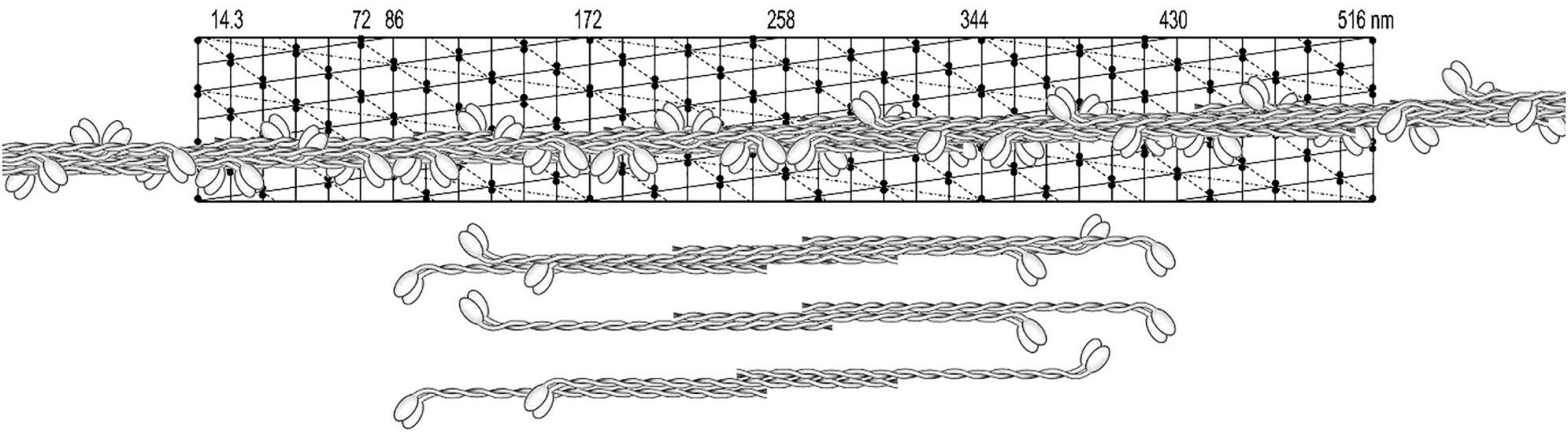
Assembly of long hexamer unit into a continuous helical strand. This unit is formed from two anti-parallel trimers shown below and it extends over two helical repeats (2 × 172 nm). Note that the polarity of the cross-bridges continuously changes from right to left because of the attaching of the two opposite ends of the two hexamers within a single combining period (6 cross-bridge crowns). For more details, see text

A supra-coiled-coil thread on the proposed helical lattice is shown in Fig. 17 with the proposed building unit. As it is shown below the lattice, the two long trimers of the kind previously proposed (Sobieszek 2016). These trimers within the hexamer unit, exhibited several kinds of long or short overlapping regions of which the shortest (of 14.3 nm) and the longest (86 nm) may play a role in the filament assembly (see “Discussion” section).

**Fig. 18:**
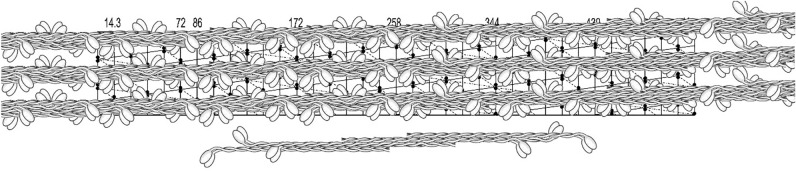
Three helical strands with the corresponding hexamer building unit. Note that the targeted row variability of the cross-bridges is now correct although it may not be readily recognized along each strand. It is important for this figure as well as all figures above that the angle of a single head pair represents only the direction in which these heads would be pulling the thin actin filaments, but not necessarily the myosin filaments’ polarity

In contrast to that shown in Fig. 14, the opposite polarities of any cross-bridges (not the pair of myosin heads) are clearly seen. Figure 18 shows a complete filament assembly, in which the three supra-coiled-coil threads were incorporated. There are three cross-bridges at each 14.3 nm crown, covering the entire width and length of the lattice. In Figs. 16, 17, 18, the angle of a single head pair represents only the direction in which these heads would be pulling the thin actin containing filaments. The horizontal angular separation of the heads was 120° as in most other muscle systems, and this angle is changing by 20° along a helical strand (see below). It is up to future studies to investigate further this model using high-resolution images of these filaments and analyze them with more advanced approaches, which are available now.

Polarity of the filaments is the last parameter that was incorporated into the proposed model and it is the most controversial one. As all the actin filaments are polar, the myosin filaments have to incorporate some kind of bipolarity to interact with each other in order to develop tension. Intuitively, the key solution suitable for smooth muscle has been proposed already earlier in the form of a bipolar building unit (Sobieszek 1972) and the row polarity (Hinssen et al. 1978). This was reinforced by our analysis of the optical diffraction patterns which we extended to the FFT of the EM images. However, these transforms were informatively poor and practically did not provide useful information except a confirmation of the 14.3 nm repeat. More informative was the FFT application for testing our models, how different forms of cross-bridges may affect the diffraction patterns.

### Targeted row variability and its analysis of the model by Fourier transform

As shown in Fig. 18, a filament assembled from a bipolar hexamer made of two asymmetric trimers fulfilled all the necessary criteria for smooth muscle function including the targeted row variability feature. Thus, we had a tool to check how the polarity of the cross-bridges would modify or influence the FFTs. Using ImageJ software, these transforms were obtained for numerous cross-bridge shapes including a very realistic one (see Winkelmann et al. 1991), each for the three possible arrangements: the left and right oriented heads as well as the one for the targeted row variability (mixed) orientation.

**Fig. 19:**
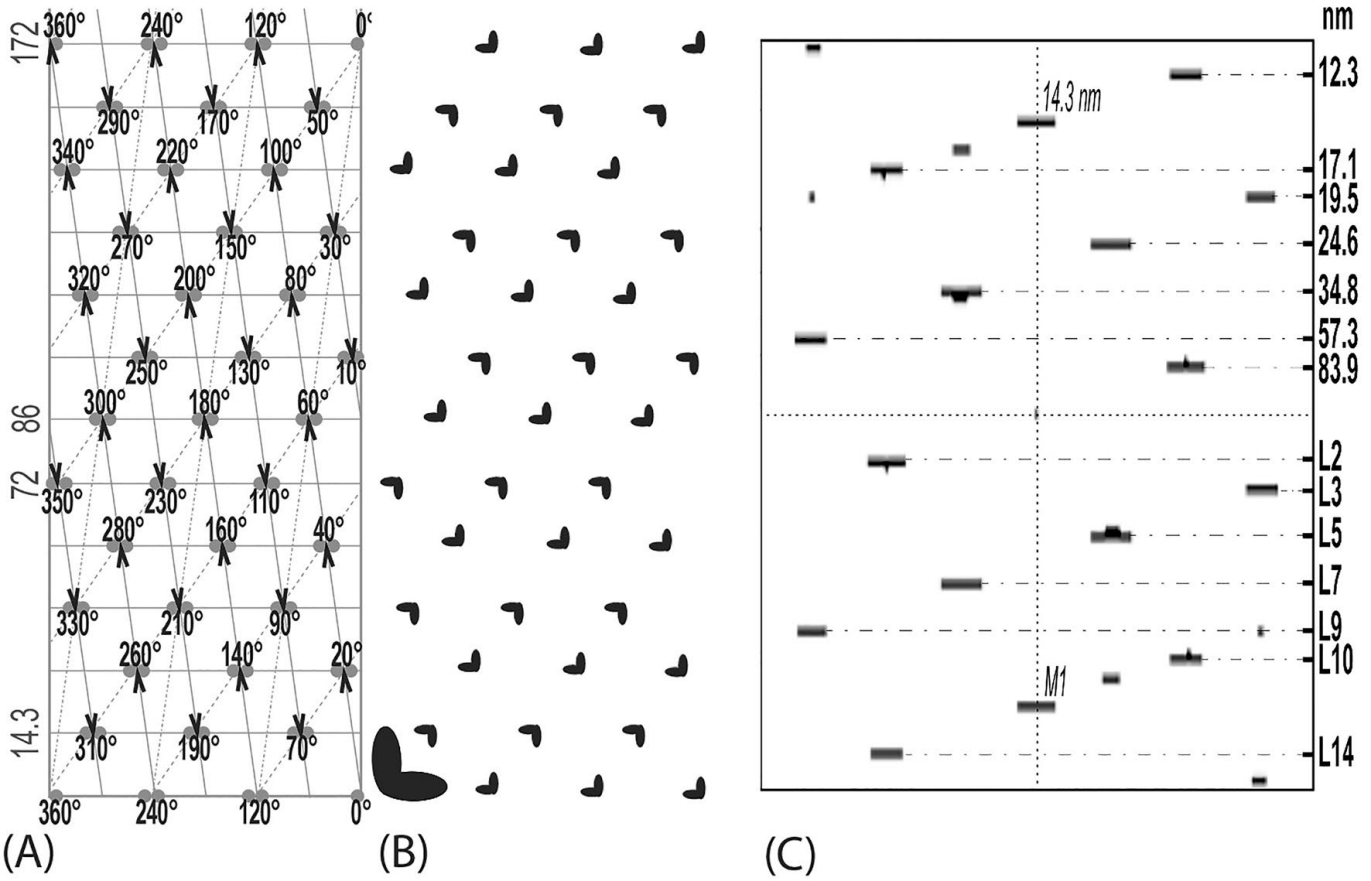
Proposed supra-coiled-coil assembly model of the vertebrate smooth muscle. **A** Arrangement of myosin cross-bridges on the helical lattice with their polarity (arrows; i.e.: TVR) and horizontal orientations. **B** The distribution of the cross-bridges on the mask used for obtaining Fast Fourier transforms (FFT). The point of attachment relative to the lattice influenced the FFTs. **C** The transform was obtained from a longer lattice (516 nm) and with the cross-bridges in form of “bunny ears L 90°”. Their inner corner was always placed at the lattice crossing points. The shape and positioning of the cross-bridge on the lattice influenced greatly the symmetry of the FFT and, to a lesser extent, even the repeat. The three (or six) strands helix is characterized by 172 nm repeat and 2 × 14.3 nm stagger of the building units (myosin hexamer composed of two trimers). Within a repeat, there are 2 × 18 adjacent rows of the cross-bridges, which are oppositely polarized and 12 crowns of myosin heads (3 pairs of heads at each of the 14.3 nm repeats). The values of the layer lines obtained are given at the right axis. For more details see “Discussion” section

As it is shown in Fig. 19, the LL distributions in the FFTs obtained from the targeted row variability or mixed polarity were symmetrically balanced, at the left and right of the meridian. (These transforms or related optical diffraction patterns are ideally asymmetric when obtained from one side of a helix, but with the contribution from both sides, they are ideally symmetrical.) The LL distributions of the transforms, obtained from either the left or the right polarity, were always asymmetrical, representing a mirror image of each other.

We examined dozens of FFTs obtained from three different shapes of cross-bridges: dots and “bunny ears” (or heart shaped) as well as the most realistic ones deduced by Winkelman in 1991 (Winkelmann et al. 1991). We observed that there are two kinds of weaker reflections accompanying the main 14.3 meridional one. The first one (of very weak intensity or even absent) was very close to the main 14.3 nm (not shown) and the other of lower intensity, was in the first left column of the reflection, next to the 14.3 nm one (see Fig. 19C). The position of the first one depended on the symmetry of the “mass” (area of black color) and it was exactly in line with the main meridional one, as observed by Craig (Xu et al. 1997). The occurrence of the second reflection depended on the angular “mass distribution”; as in 1972 illustrated in Plate XII by Small and Squire (1972).

We established that the more intense reflection was only present in the case of the mixed or targeted row variability polarity and it was absent in the FFT obtained from singly polarized cross-bridges, neither left nor right. Thus, it is apparent that the previously described characteristic features of the “face” (ribbons) or “side” (square filaments) polarities (see “Introduction” section) have been wrongly interpreted. The presence of the 86 and 57.3 or even the 34.4 nm LLs in the FFT of the configuration as shown in Fig. 19C, clearly supported the proposed 172 nm repeat. At the same time, some LLs obtained by FFTs from the same helical lattice, often indexed better with a longer or shorter repeat. No definite conclusion could be made from the presence of lower range LLs at about and below 36 nm such as the one at 24.6 nm. These two LLs are indicative of a 72 nm repeat that has been proposed previously (Sobieszek 1972, 1977a), which is characteristic for the molluscan smooth muscle (Sobieszek 1973). This is not surprising as the myosin filament interacts with the polarized partner, the actin filament, which exhibits the same 72 nm repeat in all types of the living cell.

## Discussion

### History revisited

In the early 70s, our understanding of the fine structures of smooth muscle was poor and lagged behind that of skeletal muscle for various reasons. At that time, AS was working on the structure of invertebrate smooth muscle and found himself in the middle of a controversy concerning the fine structure of myosin elements, specifically whether they are made of helically arranged subunits or in a form resembling flat ribbons. With some luck an excellent preservation of the structure of molluscan muscle (ABRM) in the relaxed state has been obtained for EM (Sobieszek 1973). This was due not only to the X-ray controls of the samples for final EM, but also for the inclusion of low concentrations of DMSO during initial fixation of the muscle. Encouraged by his colleague Dr. Small from the same group, AS processed mammalian taenia coli muscle in exactly the same way. This has resulted in the uniform preservation of a protein structure in the muscle that exhibits high degree of order. The well-preserved structure can be seen at higher magnifications, with actin filaments arranged in regular lattices, similar to that of the ABRM muscle (Sobieszek 1973).

In contrast, the cross-sectional profiles of the myosin filaments were not uniform, some being round with a hollow middle due to the myosin heads projecting away from the filament backbone, but also the profile was often seen as a rectangle, suggesting a ribbon-like structure for the filament, similar but not as wide as the ribbons observed by Small and Lowy (Lowy and Small 1970; Small and Squire 1972). Thus, preservation of such myosin filament fine structures could not resolve the existing controversy about the shape of the myosin contractile elements in smooth muscle, especially in view of the inconclusive X-ray diffraction data on the living muscle (Lowy et al. 1973; Shoenberg and Haselgrove 1974).

Importantly, even decades later, this controversy remained in spite of the further and more advanced X-ray studies on living muscles. The intensity of the relevant 14.3 nm reflection does not correlate with the number of assembled filaments (Watanabe et al. 1993). Their assembly appears to be enhanced during contraction for some muscle types, but not for others (Suzuki et al. 1978). Thus, it is apparent that there are additional factors involved in the filament assembly process. One of them is the phosphorylation of myosin by the MLCKase (Craig et al. 1983), which is tightly associated with these filaments (Sobieszek 1990). As illustrated in Fig. 13, the loose and/or tight packing of the filament backbone may be physiologically relevant, in addition to telokin, which has been shown to modulate myosin phosphorylation (Sobieszek et al. 2005a, b) and the assembly process (Sobieszek 2022, in press).

### Present understanding of the structure

The inconclusive X-ray data on the filaments’ structure prompted us to examine our old optical diffraction data obtained from the EM images of these filaments; including more recent ones from pig stomach muscle. These filaments appeared particularly interesting because of their apparent excellent preservation of the cross-bridges (e.g., see Fig. 10). With such fine visible details, it was clear that these filaments were round and not square in shape as it has been concluded by others (Craig and Megerman 1977; Xu et al. 1996, 1997). The mode of assembly is embedded in the sequence of the myosin, and this has to be helical like in all muscle types. Thus, it is unlikely that evolution has led to a less ordered square shape, decreasing their optimal interaction with the actin filaments.

**Fig. 20:**
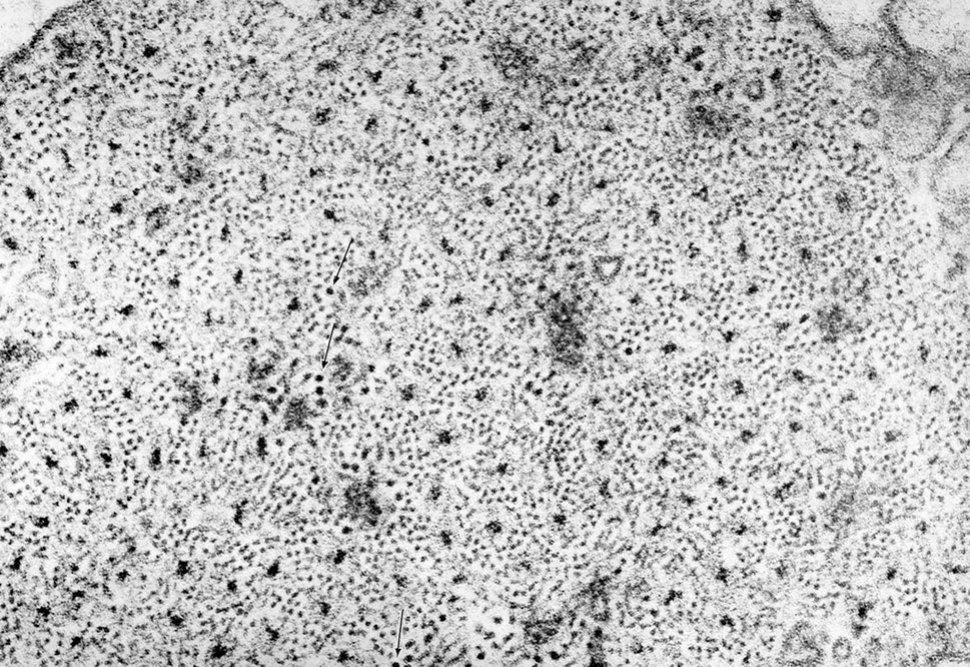
Regular arrangement of thick and thin filaments of rabbit portal-anterior mesenteric vein presented already in 1972 (Somlyo et al. 1973). The authors named the profiles of the myosin filaments as “rosettes” and they are comparable to the larger ones shown in Fig. 4 of the ABRM, in which the myosin heads or cross-bridges could often be identified. The filaments were clearly not of square profile as concluded by Craig and his colleagues. We thank Prof. Avril V. Somlyo for this EM image, which was included with the permission of the Royal Society of London. Magnification: ×143,000

Importantly, in a parallel EM study, Somlyo and his colleagues (1973) presented a uniform preservation of myosin filaments within a smooth muscle cell (see also Fig. 20). This contrasts with our preservation shown in Fig. 3, in which some narrow ribbon like structures were present, both obtained under relaxed conditions. At lower magnification, our preservation of the tissues (Fig. 1), and that of Somlyo group, was equally uniform. In both cases, the cells were more densely packed, and their extracellular space was filled up with collagen fibers. Preservation of these vertebrate tissues during contraction as a whole is difficult or nearly impossible, except perhaps during smooth muscle constriction. This state is analogous to the catch state of the molluscan smooth muscles and, as in the vertebrates, the arrangement of the thick filaments under the catch state remains to be established.

### Possible role of myosin rod and its flexible subfragment in the assembly.

The initial indication of a unique assembly of the smooth muscle myosin came from the assembly of the myosin rods or, as we call them, the “javelins” (due to the tapered ends) obtained by proteolytic digestion of myosin (Sobieszek 1977a). Significantly, the one-third shorter fragment of the rod, so-called LMM, does not form filaments but paracrystals with regular striation, resulting from the 14.3 nm repeat; thus, the flexible myosin sub-fragment 2 (SF2) appeared to be not involved in the filament formation (Gundapaneni et al. 2005). Optical diffraction patterns of these javelins provided the initial clues on the supra-coiled-coil arrangement and additionally indicated their flexibility during packing of the filament’s backbone. The slightly tilted rods may result from the inter-coiling around each other, making the suggested tight packing with the flexible SF2 fragment in the inner circle of the rod possible (Miroshnichenko et al. 2000). Independently, the contribution of the SF2 fragment to the backbone was observed in the X-ray diffraction study for skeletal muscle (Yu et al. 1985).

The assembly and plausible helical parameters of the smooth muscle myosin filaments have been described and discussed in several previous publications by AS and his colleagues (Hinssen et al. 1978; Small and Sobieszek 1980; Sobieszek 1977a, 1972). In these publications, the authors describe several additional features related to the assembly of these filaments such as a bipolar building unit (myosin dimer), which would form our three-strand continuous supra-coiled-coil helix. The important feature established previously is the absence of a bare zone characteristic of the skeletal muscle, and instead, there are two smooth edges without the cross-bridges at opposite ends, at which the filaments could elongate. Because the ribbons’ features fit well to the smooth muscle performance, Craig and his colleagues have used them in an alternative, non-helical model, in which the filaments are rectangular in cross-sectional profile with polarized cross-bridges at each side, and each has a cross-bridge-free smooth edge at the end (Craig and Megerman 1977; Xu et al. 1996). Such a structure has not been observed in any other muscle type. In contrast, the supra helical architecture of the myosin filaments appears to be universal for all types of muscles. Nevertheless, the square and non-helical form of these filaments appears to be generally accepted in the smooth muscle field (Squire 2009; Wang et al. 2021).

The short rod assemblies in the form of large arrowheads of the kind shown in Fig. 6 most likely represent looser javelins wrongly attached, and as a result elongating in the same direction, but with two angles differing by about 14°, i.e., the 2 × 6°–7° tilt of the meridional reflection observed in the optical diffraction patterns. Most interesting was the formation of the network of “threads” made up of the rods after their solubilization at low pH and the subsequent slow neutralization of the pH (see Fig. 8). Intact myosin denatures under such a treatment and forms a head-to-head aggregation (Sobieszek 1977a), but for the heat stable rods, the aggregation induced by pH neutralization apparently facilitated a long thread formation. This would be consistent with the formation of lyotropic liquid crystals by long rod-like macromolecules (Yu et al. 1997), and it has been noted as a “honey-like consistency pellet” during the first successful purification of smooth muscle myosin (Sobieszek 1977a).

### Interpretation of the optical diffraction patterns and the proposed model

Optical diffraction patterns from the javelins and their appearance indicated a regular supra-coiled-coil arrangement of myosin rods while the presence of the LL reflections at about 87 and 58 nm were consistent with the longer 172 nm repeat and not the shorter one at 72 nm previously proposed (Sobieszek 1977a, 1972). In contrast to the LMM paracrystals, this supra-coiled-coil arrangement, together with the tilted myosin meridional reflections, indicated a strong contribution of the flexible neck region of myosin into the filament’s structure. The presence of these two LL reflections was important because their values were less variable, therefore more exact, in the determination of the repeat of the helix. Significantly, it was also the first direct indication of their supra-coiled-coil structure.

In contrast to the javelins, analogous patterns obtained from myosin filaments were richer in number of reflections but more difficult to interpret because of their greater variability in the values of repeats or related spacing obtained from their optical diffraction patterns. In general, the relatively intense LL reflections such as the one at 24 nm would be consistent with the 72 nm as well as the 172 nm repeats. Another LL at about 34 nm fitted better to the longer 172 nm repeat, because when present it was more intense than the 24 nm one. A weaker reflection at about 19 nm fitted also well to the longer repeat, because at this range it was clearly different from the 24 nm one, characteristic of the shorter 72 nm repeat.

On this basis and in spite of the variability, which in a way is expected due to the flexibility of the cross-bridges, we concluded that the 172 nm repeat corresponds to the supra helical nature structure of the smooth muscle myosin filaments. We propose that this is a three-strand helix characterized by a 516 nm pitch and 2 × 14.3 nm residue translation. At the same time, and as it is apparent from the long 516 nm helical lattice (see Figs. 14, 15, 16, 17, 18), the head distributions were similar in the short (72 nm) and the long (172 nm) repeat. Nevertheless, after 5 or 6 of their respective repeats (at 430 nm), the position of the heads precisely coincided.

With the establishment of more accurate helical parameters (see above), together with the newly established building unit, our next goal was the elucidation of a more exact 2D-packing of the myosin within a filament and its presentation in the form of a detailed architectural model. This new model should include our previously published data, it should incorporate essential features of the smooth muscle contractile apparatus, and it should be consistent with the well-established structural details of other muscle types. Specifically in relation to smooth muscle, the proposed helical architecture appears to be consistent with the model of mixed polarity of the cross-bridges or, as we call it, the targeted row variability (see above). The model also predicts the elongation of these filaments at their two opposite ends as well as an absence of the bare zones.

Our model and its proposed large hexamer building unit explains the difficulties of the smooth muscle preparations for EM examination (see Small and Sobieszek 1980), as well as the formation of the artificial myosin ribbons in earlier studies. No doubt, these are formed in the muscle fibers under extensive stretch and relaxed conditions, before fixation for EM. It is interesting to note, that Somlyo has given such an explanation for the ribbon formation already in 1973 (Somlyo et al. 1973), where an excellent preservation of rabbit portal-anterior vein does not show even a single ribbon (see above Fig. 20). We suggest that the ribbons were formed from the already flat, coiled threads made of the hexamer units, which do not build filaments under relaxed conditions. These were compressed together when stretched before and during fixation in between groups of actin-lattices. It is unlikely that they are formed by side-by-side aggregation of the filaments as suggested by Small (1977).

### The tetramer building unit and possible analogy to the structure of other fibers

In a recent optical diffraction study, AS showed that, in spite of its high solubility at low ionic strength, the trimer is a predominant form of the smooth muscle myosin even at high, unphysiological ionic strength conditions (Sobieszek 2016). This has been demonstrated by dynamic light scattering measurements, in which myosin solution is passed through a size exclusion chromatography column, connected to a set-up measuring light scattering with a simultaneous calculation of the molecular weight (MW) and the hydrodynamic radius (R_h_). Therefore, a trimer but perhaps not a hexamer should be considered as the filament’s basic building unit. Such an approach cannot be applied to larger assemblies such as the filaments themselves, which are in the form of fine suspensions. Fortunately, the particle size of the filaments was evaluated by the static light scattering approach in which the intensity of the scattered light at different angles of a solution or fine suspension is measured. With the known size of the tetramer, a particle size corresponding to a hexamer has been identified (Sobieszek 2022, in press). Under roughly physiological conditions, aggregates of myosin of this size have been demonstrated by Trybus and Lowey (1987) and this has been confirmed recently by Korn and his colleagues (Liu et al. 2020).

In principle, the head-to-head assembly and elongation of the filaments, which enable the targeted row variability would also be possible for an 86 nm repeat. However, as such, it was not considered based on the common presence of the 58 nm LL. The proposed filament assembly exhibited an approximate five-fold symmetry in which fibration and/or formation of ribbons is facilitated (Sadoc and Rivier 1999). Such assembly can be visualized by a torus made by piling up pentagonal antiprisms in form of two fivefold symmetry tori in which the upper and lower vertices slightly discretized (Sadoc 2001). This kind of supra helical threads of 72 nm repeat may produce the elongation of the repeat of 172 nm during the first steps of the filaments’ assembly. This would be expected for a non-integral Boerdijk–Coxeter type helical assembly. Further investigations are definitely needed to confirm this conclusion because in principle the long antiprism of such assembly would correspond to our elongated trimer, and the formula describing the volume of such compact, elongated antiprisms has been recently derived (Abrosimov and Vuong 2018).

Significantly, there is an analogy in all these fibers in the way of how they assemble; from coiled-coil building units (trimers and/or tetramers) to super coiled-coil structures in other fibrous organelles of various tissues. The novelty of our model is the incorporation of the much larger hexamer building unit, which is made of two coiled-coil trimers. In turn, three super coiled-coil strands of helices form the fiber or filament backbone. This type of structural hierarchy is observed in collagen (Shoulders and Raines 2009) and keratin fibers (Wang et al. 2016) as well as in desmin filaments (Goldman et al. 2012). We propose that the main difference lies in the stabilization of these fibers. Collagen and desmin fibers are stabilized via covalent crosslinking between the building units, while for the smooth muscle myosin filament such a cross-linkage is absent because this feature is essential for the proper function of the muscle where structural malleability is important. Nevertheless, a relatively rapid increase in their stability is necessary for the smooth muscle filaments during contraction or constriction. We suggest that an answer to this question may be in the proposed head-to-head mode of the filament assembly (see below).

### Problems and unsolved aspects

Under relaxed conditions, the malleable subcellular structure of smooth muscle resembles liquid crystal-like solutions in which the long rods of the myosin will have a tendency to form a liquid crystal-like solution (Yu et al. 1997). We suggest that this would be the initial form of aggregation or assembly. Further stabilization requires free energy, which could come from MgATP hydrolysis during ordered myosin phosphorylation of the myosin heads. A singly phosphorylated myosin has been routinely prepared in many of the projects carried out by AS. In his recent work, such a myosin was more readily assembled than the unphosphorylated one (Sobieszek 2022, in press). Earlier on, it has been shown that phosphorylation increases the filament assembly 25 to 50-fold (Kendrick-Jones et al. 1987).

As established by AS (see Sobieszek 1991), assembled filaments contain an endogenous CaM/MLCKase complex that is capable of very rapid phosphorylation during activation (Sobieszek 2001). Importantly, long filaments phosphorylated at a rate 2–3 times slower in comparison with that of the short ones and this rate is further reduced in the presence of telokin. Thus, the assembly mechanism appeared to be modulated by this protein, which inhibits the myosin phosphorylation rate, and not its amplitude (Sobieszek et al. 2005a, b). In our view, this is one of the main feature characteristics for the smooth muscle contractile apparatus and therefore, has to be reflected in the supra helical architecture of these filaments.

Another example of such a unique architecture has been recognized but so far is not fully understood, i.e.: that of the invertebrate smooth muscle myosin filaments. As seen in the Results, there are important similarities but also differences in the two smooth muscle types. The similarity relates to the thin, actin-containing filaments and their structure and organization, i.e.: the presence of the so-called actin lattices. The difference between the thick myosin-containing filaments is extraordinary (see Fig. 11E) because these filaments are not only extremely large (in their length and diameter) but build up with two additional rod-like proteins. The first protein is called “paramyosin” (Sugi et al. 2020) and the other one has been named “myorod” (Shelud’ko et al. 1999). As it is apparent from the former review, the backbone architecture of these large myosin filaments is still not understood, nevertheless, the supra-coiled-coil assembly of the molluscan myosin on their surfaces appears to be similar to that described in the study by AS in 1973 (Sobieszek 1973).

## Conclusion

To summarize, the supra helical architectures of smooth muscle myosin filaments are the most common form of assembly in living cells, especially in their fibrous organelles. So far, only a few have been characterized in detail, these include creatin and collagen fibers as well as desmin filaments; the last two also perform important roles in smooth muscle function. In contrast, this adaptable architecture of the myosin filaments, essential for smooth muscle function has remained controversial for decades. In the present report, we analyze our old unpublished EM data on the assembly of purified myosin and its rod sub-fragment. The analysis by optical diffraction confirmed our earlier conclusions about the supra coiled-coil structure of these filaments and made it possible to develop a detailed 2D-model of their architecture. The model not only explains and elaborates our earlier observations on the filament assembly, but also hypothesizes that it is analogous to a non-integral helix of the Boerdijk–Coxeter type (Sadoc 2001). There is however, an important difference necessary to accommodate a unique muscle feature, specifically the absence of covalent crosslinking between the building units of filaments. No doubt, further studies are needed to verify our predictions in relation to our model conclusions.

## Data Availability

The authors confirm that the data supporting the findings of this study are available within the article.
